# The trehalose pathway in maize: conservation and gene regulation in response to the diurnal cycle and extended darkness

**DOI:** 10.1093/jxb/eru335

**Published:** 2014-09-30

**Authors:** Clémence Henry, Samuel W. Bledsoe, Allison Siekman, Alec Kollman, Brian M. Waters, Regina Feil, Mark Stitt, L. Mark Lagrimini

**Affiliations:** ^1^Department of Agronomy and Horticulture, University of Nebraska-Lincoln, Lincoln, NE 68583-0915, USA; ^2^Max Planck Institut für Molekulare Pflanzenphysiologie, Potsdam (OT) Golm, Germany

**Keywords:** Maize, shade stress,, trehalose-6-phosphate, trehalose gene family, diurnal cycle,, quantitative RT-PCR.

## Abstract

Energy resources in plants are managed in continuously changing environments, such as changes occurring during the day/night cycle. Shading is an environmental disruption that decreases photosynthesis, compromises energy status, and impacts on crop productivity. The trehalose pathway plays a central but not well-defined role in maintaining energy balance. Here, we characterized the maize trehalose pathway genes and deciphered the impacts of the diurnal cycle and disruption of the day/night cycle on trehalose pathway gene expression and sugar metabolism. The maize genome encodes 14 trehalose-6-phosphate synthase (TPS) genes, 11 trehalose-6-phosphate phosphatase (TPP) genes, and one trehalase gene. Transcript abundance of most of these genes was impacted by the day/night cycle and extended dark stress, as were sucrose, hexose sugars, starch, and trehalose-6-phosphate (T6P) levels. After extended darkness, T6P levels inversely followed class II TPS and sucrose non-fermenting-related protein kinase 1 (SnRK1) target gene expression. Most significantly, T6P no longer tracked sucrose levels after extended darkness. These results showed: (i) conservation of the trehalose pathway in maize; (ii) that sucrose, hexose, starch, T6P, and TPS/TPP transcripts respond to the diurnal cycle; and(iii) that extended darkness disrupts the correlation between T6P and sucrose/hexose pools and affects SnRK1 target gene expression. A model for the role of the trehalose pathway in sensing of sucrose and energy status in maize seedlings is proposed.

## Summary

Extended darkness induces a transient increase in sugars and trehalose pathway gene expression.

## Introduction

A central feature of plant metabolism is the photosynthetic conversion of light energy into stored chemical energy. Every 24h, plants cycle from net energy production to net energy consumption. During the day, plants produce sucrose and reducing sugars used in the synthesis of amino acids, lipids, nucleic acids, and complex carbohydrates. As light energy wanes at dusk and throughout the night, the plant transitions from a net producer of sugars to a net consumer. During the night, the plant utilizes stored carbohydrates as a source of carbon skeletons and chemical energy (Baena-González *et al.*, 2007; Stitt and Zeeman, 2012). In some plants, like *Arabidopsis*, the vast majority of the stored carbohydrate is in the form of starch (Gibon *et al.*, 2009; Sulpice *et al.*, 2010, 2014), but in other plants, sugars including hexoses and sucrose can play a role in maintaining energy balance throughout the transition between light and dark. Trehalose [α-d-glucopyranosyl-(1→1)-α-d-glucopyranoside] is an important osmotic protectant in bacteria, fungi, and insects where it accumulates to high concentrations (Avonce *et al.*, 2006). Most plants accumulate only trace amounts of trehalose and its intermediates, where it is unlikely to function as an osmoprotectant (Paul *et al.*, 2008). Rather, the role of the trehalose metabolic pathway and its intermediates is to sense and communicate energy status (Lunn, 2007; Lunn *et al.*, 2014). As examples, exogenously applied trehalose altered physiology and gene expression, such as induction of the AGPase gene in *Arabidopsis* (Wingler, 2002), and resulted in increased biomass yield and water-deficit stress tolerance (Rodríguez-Salazar *et al.*, 2009; Sciences and Zeid, 2009; Ali and Ashraf, 2011). The inflorescences of the *ramosa3* mutant of *Zea mays* have significantly reduced trehalose (Carillo *et al.*, 2013) and excessive branching (Satoh-Nagasawa *et al.*, 2006). An induced increase in trehalose-6-phosphate (T6P) inhibits starch degradation in *Arabidopsis*, and changes in T6P modulate the photoperiod and flowering patterns (Wahl *et al.*, 2013).

Plants have a conserved three-step metabolic pathway for the synthesis and degradation of trehalose. In the first step, trehalose-6-phosphate synthase (TPS) catalyses the condensation of glucose-6-phosphate (G6P) and uridine diphosphate glucose (UDPG) to form T6P. Trehalose-6-phosphate phosphatase (TPP) subsequently removes phosphate to form trehalose. Trehalase (TRE) then hydrolyses trehalose into two glucose residues. Plant TPS and TPP are encoded by multigenic families, while the trehalase (TRE) gene is present in a single copy (Lunn, 2007)*. Arabidopsis* and rice genomes each encode 11 TPS genes and, respectively, 13 and 10 TPP genes (Yang *et al.*, 2012). TPS genes are divided into two classes. Class I TPS genes are generally present in a single copy, and they usually encode catalytically active TPS enzymes that have both TPS and TPP domains, with inactive phosphatase boxes. Class II TPS genes have both TPS and TPP domains but lack residues in the TPS domain needed for interaction with the substrate. Most class II TPS genes have conserved phosphatase domains; however, they do not possess TPS or TPP activity (Vandesteene *et al.*, 2010). In rice, some class II TPS proteins interact to form high-molecular-weight complexes, and a regulatory role is suspected (Zang *et al.*, 2011). All plant TPP genes are composed of a unique TPP domain with conserved phosphatase domains, and all encode functional TPP enzymes in *Arabidopsis*. Since they have similar activity but differential expression patterns, TPP genes probably have a tissue-, stage-, and/or process-specific function (Vandesteene *et al.*, 2012).

The diurnal switch from energy production to energy consumption requires a global change in gene expression and metabolic networks. In concert with the internal clock, sugar levels are a key regulator of this switch. Sugar levels fluctuate during the diurnal cycle, and sugars and circadian rhythm have an approximately equal and interactive effect on gene expression (Bläsing *et al.*, 2005). In maize, at least 10% of transcripts display circadian expression patterns, with peak expression at dawn and/or dusk in preparation for the periodic change in environment (Khan *et al.*, 2010). Not surprisingly, many diurnally regulated transcripts encode proteins involved in photosynthesis, respiration, carbohydrate metabolism, and cell elongation (Harmer *et al.*, 2000). Understanding how this switch takes place is of fundamental importance to improve crop productivity.

Plants have complex sugar signalling networks to maintain energy status regardless of photosynthetic output or growth rate (Sheen, 2010). Hexoses are sensed through hexokinase (HXK)-dependent and HXK-independent pathways (Sheen, 2010). Sucrose sensing is less well understood; however, a correlation between sucrose and T6P levels strongly suggests that one route may involve a T6P inhibitory effect on sucrose non-fermenting-related protein kinase 1 (SnRK1), a global integrator of energy balance (Polge and Thomas, 2007; Zhang *et al.*, 2009; Nunes *et al.*, 2013). When energy levels decrease due to starvation or stress, SnRK1 is activated and triggers induction or repression of ~1000 genes to switch from anabolism to catabolism, promoting survival in lieu of growth (Baena-González *et al.*, 2007). This effect on gene expression probably involves the basic region leucine zipper transcription factor 11 (*bZIP11*) (Delatte *et al.*, 2011; Ma *et al.*, 2011).

As a consequence of altered carbohydrate metabolism (Lunn, 2007), dramatic phenotypes of plants with altered expression of trehalose pathway genes include effects on flowering, embryogenesis, branching, plant stature, biomass, grain yield, and abiotic/biotic stress tolerance (Wingler, 2002; Lunn *et al.*, 2014). The role of the trehalose pathway is significant; however, the details remain unclear. Recent evidence points to T6P having a central role in sugar sensing (Paul, 2007; Zhang *et al.*, 2009; Ponnu *et al.*, 2011; Wingler *et al.*, 2012; Wahl *et al.*, 2013). Sucrose and T6P levels were correlated in *Arabidopsis* meristems (Wahl *et al.*, 2013) and seedlings recovering from starvation (Lunn *et al.*, 2006), and developing wheat grain showed a close correlation between sucrose, T6P, and SnRK1 levels (Martínez-Barajas *et al.*, 2011), suggesting that T6P can act as a signal to indicate sucrose levels (Lunn *et al.*, 2014). Recent work in *Arabidopsis* showed the ratio between T6P and sucrose to be tightly regulated and critical to maintaining homeostasis throughout the diurnal cycle and during periods of stress (Yadav *et al.*, 2014).

Most work describing T6P and trehalose in energy-sensing networks has used *Arabidopsis*, which is a reference species for dicots and for C_3_ photosynthesis. Little is known about the trehalose pathway gene structure, regulation, or role in central metabolism in the C_4_ monocot maize, although maize is a major world crop that impacts on human and animal nutrition, and is an alternative energy source. The biodiversity in maize and availability of ‘omics’ data will be synergistic tools to investigate the impact of this pathway on plant growth and development.

This study aimed to identify and classify maize TPS/TPP/TRE gene families, and to determine their response to fluctuations in sugar and energy levels throughout the day/night cycle, after extended darkness (48h) to impose an energy deficit, and during recovery from this dark treatment. Additionally, we compared starch/sucrose/hexose/T6P levels with TPS/TPP gene expression during recovery from extended darkness. A model is presented to integrate these new data into a more general view of the role of this pathway in plant growth and its response to the environment.

## Materials and methods

### Plant growth, treatment and harvest

**Fig. 1.**
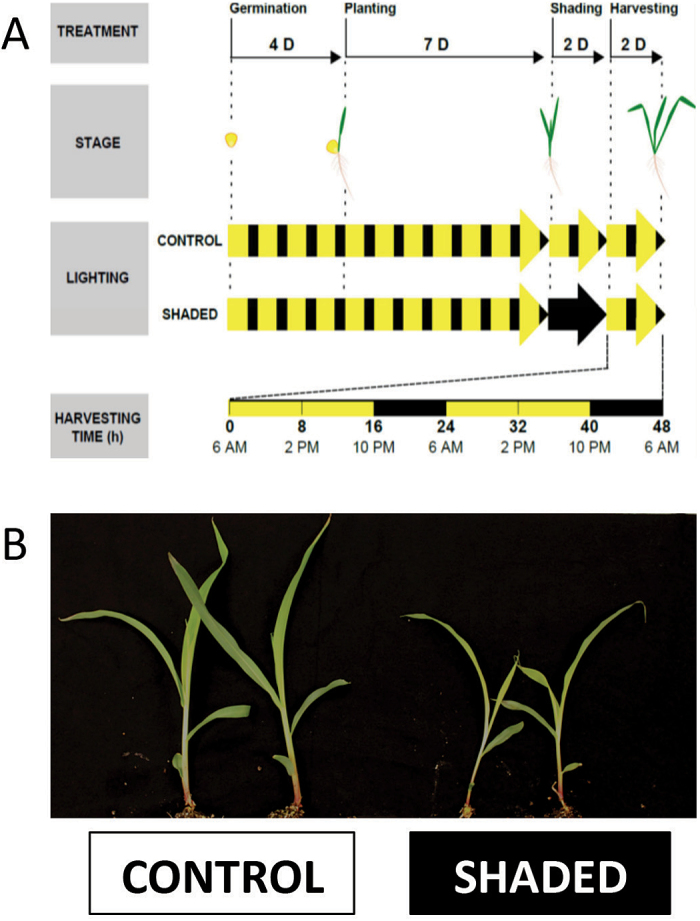
Experimental design (A) and plant phenotype after 48h of treatment (B). (A) B73 maize seeds were germinated for 4 d, planted, and cultivated under control diurnal cycles (16h day (D)/8h night (N), 220±30 µmol m^–2^ s^–1^, 24 °C, 50% relative humidity) for 7 d. The plants were then kept under the same conditions (control) or under total darkness (shaded) for 48h. They were then returned to regular diurnal cycles for recovery. Sample harvesting was done every 8h for 48h, starting at the end of the dark period when plants were still under darkness. (B) Control and shaded plants grew at different rates as a result of the treatment.

Inbred B73 maize (*Z. mays* L.) plants were used. Seeds were sterilized for 15min with 15% bleach (v/v), rinsed thoroughly with sterile water, stirred for 1min in 70 % ethanol, rinsed again, and soaked for 5min in sterile water. Seeds were then rolled in germinating paper (Anchor Paper) and germinated for 4 d in the presence of 1mM CaSO_4_ solution in a growth chamber (16h day/8h night, 220±30 µmol m^–2^ s^–1^, 24 °C, 50% relative humidity). Seedlings were planted in germinating trays containing potting mix (34% peat, 31% perlite, 31 % vermiculite, 4% soil), grown under the same conditions as described previously and watered daily with nutrient solution (20-20-20; J. R. Peters). Thirteen-day-old juvenile plants were then placed under a control photoperiod (same as above) or shaded for 48h (frame covered with a thick black fabric shading cloth, 75×75×45cm; 0 µmol m^–2^ s^–1^), and the frame was then removed to permit recovery for an additional 48h (Brouquisse *et al.*, 1998; Mutisya *et al.*, 2009). Leaf 3 (fully-expanded) was harvested every 8h for 48h starting at the end of the dark period (first time point: 6 a.m., end of the night) from plants randomly picked in the tray (Fig. 1). The centre one-third of the leaf (100–200mg) was collected into pre-chilled microcentrifuge tubes, instantly frozen in liquid nitrogen, and stored at −80 °C until use.

### Gene identification and bioinformatic analysis

Genes were identified using a name search and BLAST with *Arabidopsis* sequences in the http://www.maizesequence.org and http://bioinformatics.psb.ugent.be/plaza/databases. Predicted protein sequences were then compared with those of rice, *Arabidopsis*, and poplar (Supplementary Tables S1 and S2 at *JXB* online) using the following website to generate classification and phylogenic relationships: http://www.phylogeny.fr/ (alignment with MUSCLE, phylogeny with PhyML, and tree rendering with TreeDyn). Protein sequences were also analysed using http://myhits.isb-sib.ch/ software with the following parameters (hamap, pat, prf, pre, pfam_fs, and pfam_Is) to identify conserved TPS and TPP protein domains.

### Gene expression analysis

Frozen tissues were grounded to a fine powder using a Tissue Lyser II (Qiagen). RNA was extracted using a Trizol protocol as described by the provider with the addition of 1 µl of glycogen (Invitrogen) at the beginning. RNA samples were DNase treated using RQ1 RNase-free DNase (Promega) as recommended by the supplier and stored at –80 °C until use. RNA quantity and quality were checked using a Nanodrop 8000 spectrophotometer (ThermoScientific) and electrophoresis on a 1.2% agarose gel, respectively.

Reverse transcription (RT) was performed on 1 µg of total RNA using a SuperScript III First-Strand Synthesis Supermix kit (Life Technology) with random hexamer primers. RT quality and absence of genomic DNA contamination was then checked by semi-quantitative PCR using 5 µl of cDNA at a 1:100 dilution in a final volume of 25 µl using GoTaq® DNA Polymerase (Promega). ZmEF1-1α primers (forward: 5′-AGACTCACATCAACATTGTGGTCAT-3′, reverse: 5′-GT TGT CAC CT TCAAAACCAGAGATT-3′) were designed around an intron. For real-time RT-PCR, 5 µl of cDNA at a 1:50 dilution was used for reactions with SsoAdvancedTM SYBR® Green Supermix (Bio-Rad) and 167nM primers (Supplementary Table S2) in a final volume of 15 µl. Real-time amplification was performed in the LightCycler® 480 II (Roche) using the following program: 30 s at 95 °C; and 45 cycles of 5 s at 95 °C, 30 s at 60 °C, and 10 s at 72 °C. A melting-curve analysis was performed for 5 s at 95 °C, followed by 5 °C increments from 65 to 95 °C. For each time point and three biological replicates, quantitative PCR was performed three times. Six reference genes (Supplementary Table S2) were tested and three were selected using the Genorm software (Vandesompele *et al.*, 2002). Relative gene expression was then calculated using the formula of Hellemans *et al.* (2007). Primer efficiency was determined using the method described by (Pfaffl (2001).

### Carbohydrate metabolite analysis

Frozen tissues (20–100mg) were weighed and ground for 30–60 s while frozen using a Tissue Lyser II (Qiagen). Sugars (sucrose, fructose, and glucose) were then extracted following the method of Lunn *et al.* (2006) using lactose as an internal standard. Starch was extracted from the pellet generated during the extraction of soluble sugars,and quantified by analysis of glucose resulting from hydrolysis (Supplementary Methods S1 at *JXB* online). Samples were analysed with a high-pressure capillary ion chromatograph system (ICS-5000, PA-20 column; Thermo Scientific Dionex) using a 1 µl injection volume and 45mM KOH eluent. Sugar peaks were identified in comparison with known sugars, and data were analysed using the formulae described in Supplementary Methods S2 at *JXB* online. The method of Lunn *et al.* (2006) using anion-exchange liquid chromatography, linked to tandem mass spectrometry, was used to quantify T6P.

### Statistical methods

Pearson correlation coefficient matrices between transcripts, sugars, and transcript versus sugars were determined were computed using the stats package from R software version 3.0.1 (R Core Team, 2013, http://www.r-project.org/). The average of three biological replicates was used to perform tests. Heat maps were then generated in MS Excel using conditional formatting functions.

Student’s *t*-test was used to compare differences between control and shaded condition for each time point. Analysis was performed using the following website: http://www.physics.csbsju.edu/stats/t-test.html. Significant differences with a value of *P*<0.05 are indicated by an asterisk.

## Results

### Classification of maize TPS/TPP genes

We identified 14, 11, and one genes predicted to encode for TPS, TPP, and TRE maize enzymes, respectively (gene accession numbers in Table S1) from maize genome databases (http://www.maizesequence.org and http://bioinformatics.psb.ugent.be/plaza/). The maize genome also encodes several genes with truncated TPS/TPP domains, named TPS-like or TPP-like. These are unlikely to be functional trehalose pathway enzymes based on domain analysis using the MyHits tool; therefore, we did not investigate them further.

**Fig. 2.**
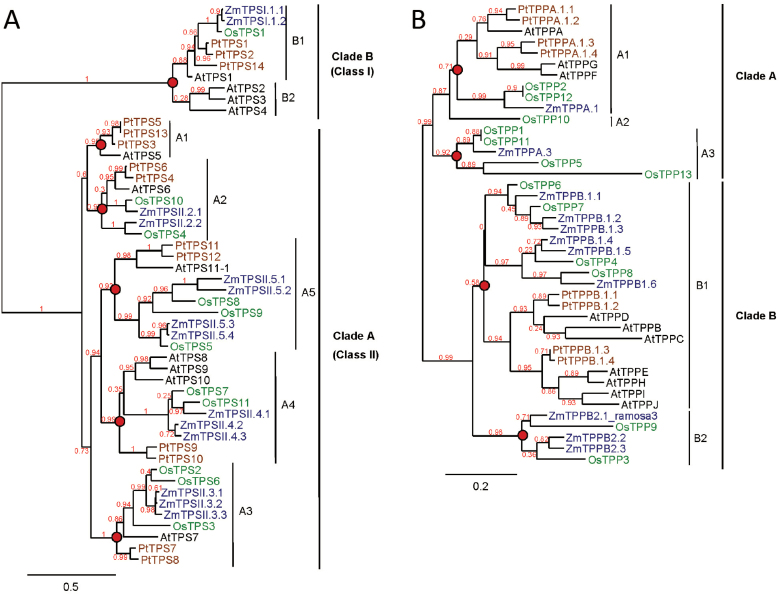
Phylogenic trees of TPS and TPP genes from *Z. mays*, *Arabidopsis thaliana*, *Oriza sativa*, and *Populus trichocarpa*. A phylogenic tree was developed using predicted protein TPS (A) and TPP (B) sequences from maize (blue), *Arabidopsis* (black), rice (green), and poplar (brown) identified from genomic databases (http://www.maizesequence.org and http://bioinformatics.psb.ugent.be/plaza/). Common ancestors before the split between monocots and dicots are indicated by red circles. Clades and subclades are indicated and bootstrap values are shown in red. Bars, amino acid substitutions per site.

As described by Yang *et al.* (2012), TPS genes were divided into two clades: clade B included all class I TPS genes while clade A included all class II TPS genes. Clades B and A subdivided into two and five subclades, respectively, corresponding to groups with common ancestors before the split between monocots and dicots. Class A was found in all dicots and subclade B2 was specific to *Arabidopsis*. Maize encoded two class I TPS genes (clade B) and 12 class II TPS genes (clade A), named according to their position in the phylogenetic tree (Fig. 2A). All maize TPS proteins included both a TPS and TPP domain. Among the class I TPS genes, clade B1 contained the functional TPS from rice and *Arabidopsis* and both maize TPS class I genes. *ZmTPSI.1.1* (previously named *ZmTPS1*) encoded a functional TPS enzyme and had all conserved TPS motifs (Table 1, Supplementary Fig. S2 at *JXB* online) (Jiang *et al.*, 2010). Structurally, *ZmTPSI.1.2* was a truncated version of *ZmTPSI.1.1* and was missing amino acids required for substrate binding. This gene is therefore unlikely to encode a functional TPS enzyme. Interestingly, all class I TPS proteins lacked the first phosphatase motif required for the catalytic activity, although they possessed a full TPP domain. Maize class II TPS genes were composed of subclades A2–A5 with *ZmTPSII.3.1*, -*3*.*2*, and *-3.3*; *ZmTPSII.4.2* and *-4.3*; *ZmTPSII.5.1* and *-5.2*; and *ZmTPSII.5.3* and *-5.4*, respectively. Maize class II TPS enzymes had a substitution of arginine with aspartic acid in the UDPG phosphate-binding pocket (Table 1). Most maize class II TPS displayed substitution of three to four amino acids in the UDPG- and G6P-binding sites, while class II TPS genes belonging to clade A5 showed a higher number of substitutions in the UDPG-binding site but had a highly conserved G6P-binding site.

**Table 1.**
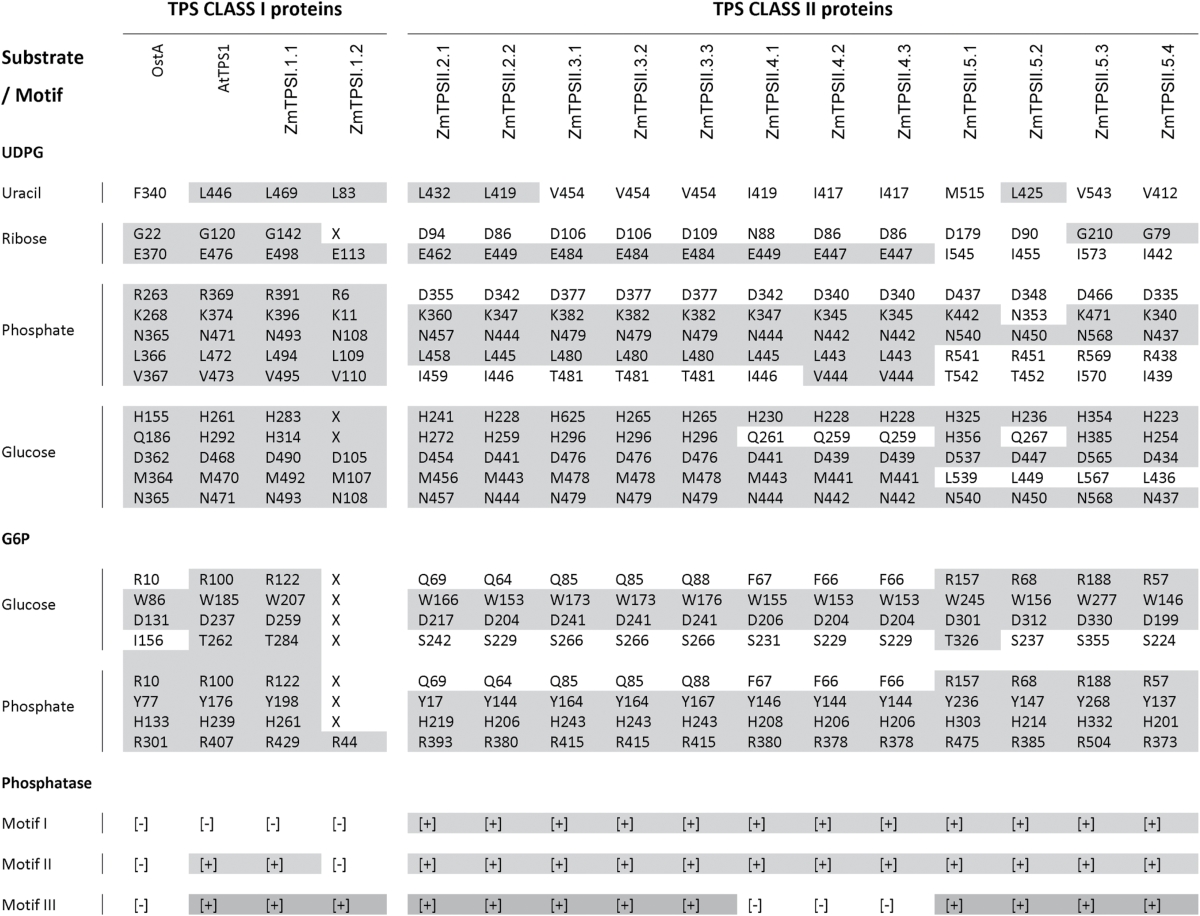
Conserved amino acids required for TPS and TPP activity in TPS predicted proteins from E. coli, Arabidopsis and maizeResidues involved in substrate interaction of the TPS domain are designed by a letter associated with a number indicating their position (Gibson *et al.*, 2002; Vandesteene *et al.*, 2010). Deletions are represented by a X. Presence (+) or absence (–) of the three phosphatase motifs required for the activity in the TPP domain is also indicated (Avonce *et al.*, 2006; Lunn, 2007). Conservation of residues or motifs required for TPS and TPP activity is highlighted by shading.

The TPP family also had two clades that were divided into three and two subclades (Fig. 2B). Subclades A1 and B1 included genes from both monocots and dicots, while other subclades were specific to monocot species. The position of the maize TPP genes within subclades was used for their nomenclature. TPP genes mainly evolved through duplication events after the monocot/dicot split or even after speciation, in contrast to TPS genes. All maize TPP genes displayed three conserved motifs required for TPP activity: (i) DXDX(T/V)(L/V/I); (ii) (S/T)(GX) in an hydrophobic context; and (iii) K(X)16–30(G/S)(D/S)XXX(D/N) (Table 1, Fig. S2) (Avonce *et al.*, 2006; Lunn, 2007).

### Maize TPS/TPP and SnRK1 targets gene expression

**Fig. 3.**
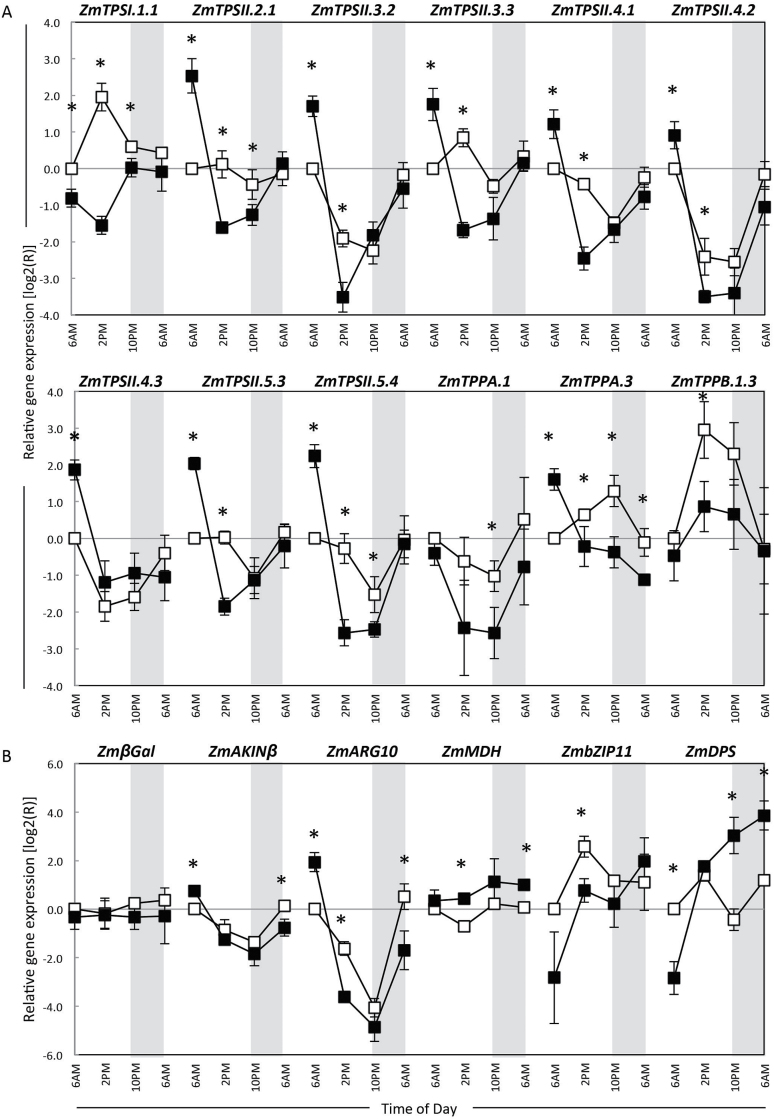
Relative gene expression for selected maize TPS, TPP, and SnRK1 putative target genes in control and shaded seedlings. Leaf tissue were collected from the V3 stage control (16h day/8h night, open squares) and shaded (48h, filled squares) plants. Sampling was done every 8h for 24h starting at the end of the night or extended shading period (recovery phase). TPS and TPP transcript levels were specifically measured using quantitative RT-PCR. Transcript levels are expressed relatively to the first time point of control treatment. Expression was normalized to the geometric mean of stably expressed genes: *ZmPP2AA2-2* and *ZmCACS*, and transformed using a log_2_ function. Data are presented as means±standard error (SE) of independent biological samples (*n*=3). Genes are grouped in three classes (A, B, and C) based on their response to regular circadian cycles (open squares).

Maize TPS/TPP gene expression was characterized throughout the regular diurnal cycle and during the recovery from 48h of extended darkness. In plants with regular diurnal cycles, gene expression patterns were quite varied among TPS/TPP genes and putative SnRK1 targets (Fig. 3). Expression of the catalytically active *ZmTPSI.1.1*, *ZmbZIP11*, and *ZmTPPB.1.3* increased throughout the morning, peaking at 2 p.m., and then decreased in the late afternoon and night. Most class II genes and *ZmTPPA.1* had their highest transcript levels at the end of the night period and decreased throughout the day. Several SnRK1 target genes were selected as indicators of a possible SnRK1 activity. We looked at the expression of some targets shown to be upregulated (βGal, AKINβ, and ARG10) or downregulated (MDH, bZIP11, and DPS) by SnRK1 in *Arabidopsis* (Supplementary Fig. S3D and E at *JXB* online) (Baena-González *et al.*, 2007; Usadel *et al.*, 2008). As with the class II TPS genes, SnRK1 inducible transcripts were highest at the end of the night period and SnRK1-repressible transcripts were lowest. *ZmTPSII.2.1*, *ZmβGal*, and *ZmMDH* showed no significant change in transcript levels throughout the day/night period.

In plants recovering from extended darkness, all of the class II TPS genes had significantly higher transcript levels at the end of the dark period compared with control plants at the end of an 8h night. Transcript levels fell between 8 and 16h in the first light period after extended darkness (2 and 10 p.m.) and returned to normal levels by 24h after shading ended. Dark stress resulted in reduced expression of *ZmTPSI.1.1* at a time it normally peaks during day/night cycle, and then returned to cycling similar to the control plants. A similar but attenuated pattern was observed for *ZmTPPA.3* and *ZmTPPB.1.3*. Among the putatively upregulated SnRK1 targets, two responded accordingly: *ZmAKINβ* and *ZmARG10* were both decreased during the day and increased at night in control plants (Fig. 3). These genes were induced by extended darkness and then repressed during the day during recovery, similarly to all class II TPS genes tested. Among the putatively downregulated targets, two, *ZmbZIP11* and *ZmDPS*, responded as expected: under extended darkness they were strongly repressed and then induced during recovery.

To determine if genes were regulated in a similar fashion, we determined their coefficient of correlation in control conditions and in dark-treated plants in the 24h following the treatment (Table 2). In control conditions, expression of some class II TPS genes positively correlated to each other, while most of them negatively correlated with *ZmTPPA.3* expression. Most of class II TPS genes positively correlated with upregulated targets of SnRK1 (*ZmAKINβ* and *ZmARG10*). Other correlations were not as clear. In dark-treated plants, correlations between gene expression were much more obvious. All class II TPS transcripts were positively correlated to each other, to *ZmTPPA.1*, and to SnRK1 upregulated targets. In contrast, their expression was negatively correlated to *ZmTPPB1.3* expression and negatively correlated with putative SnRK1 downregulated targets (*ZmbZIP11* and *ZmDPS*). Expression of *ZmTPSI.1.1* positively correlated with *ZmMDH* in dark-treated plants.

**Table 2.**
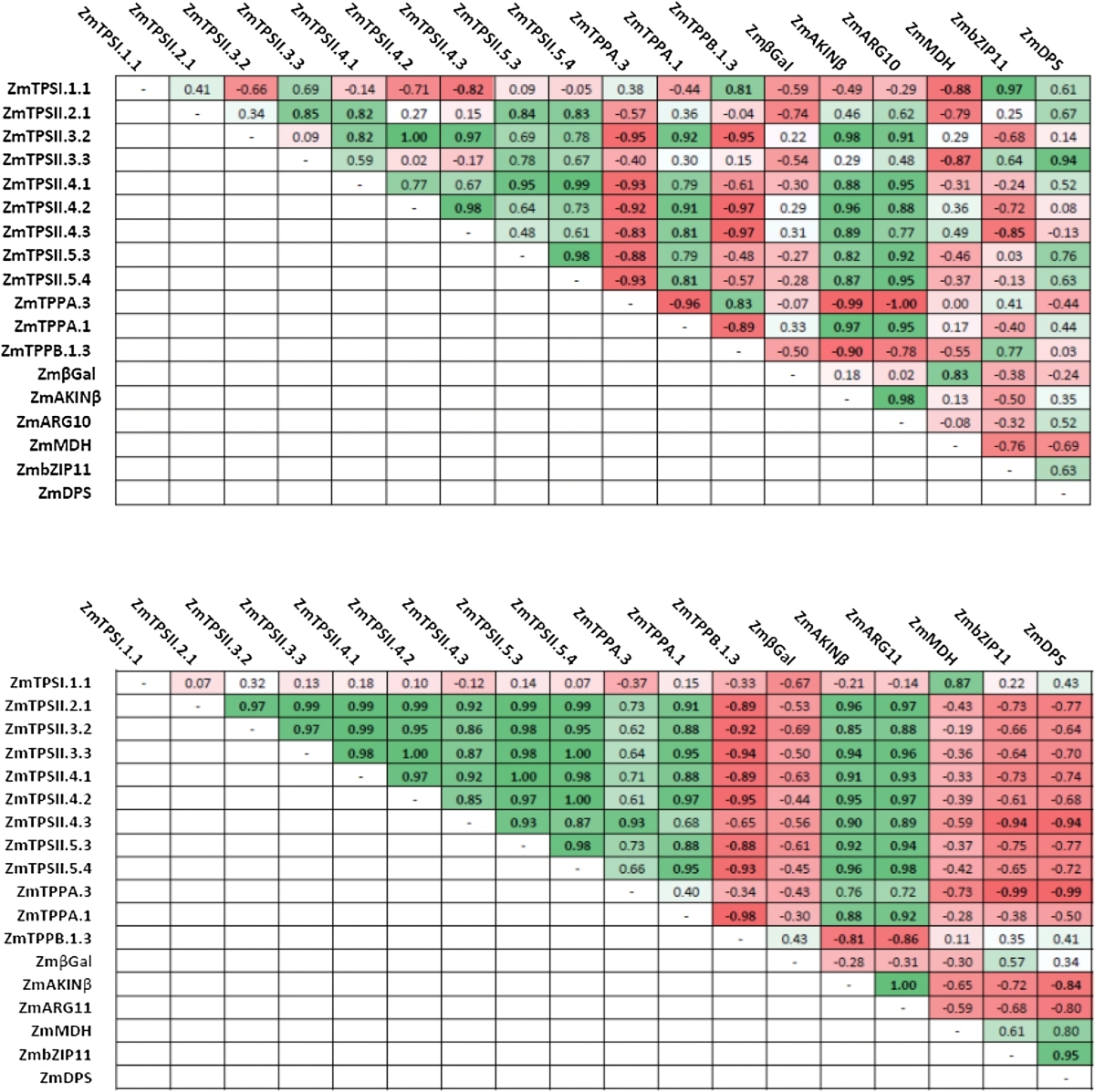
Correlation coefficient between TPS/TPP and SnRK1 target transcriptsCoefficients of correlation were determined over 24h after control (top) and dark (bottom) treatment using a Pearson comparison test (*n*=3). Positive and negative correlations are indicated in shades of green and red, respectively.

### Soluble sugars and starch in cycling and dark-treated plants

**Fig. 4.**
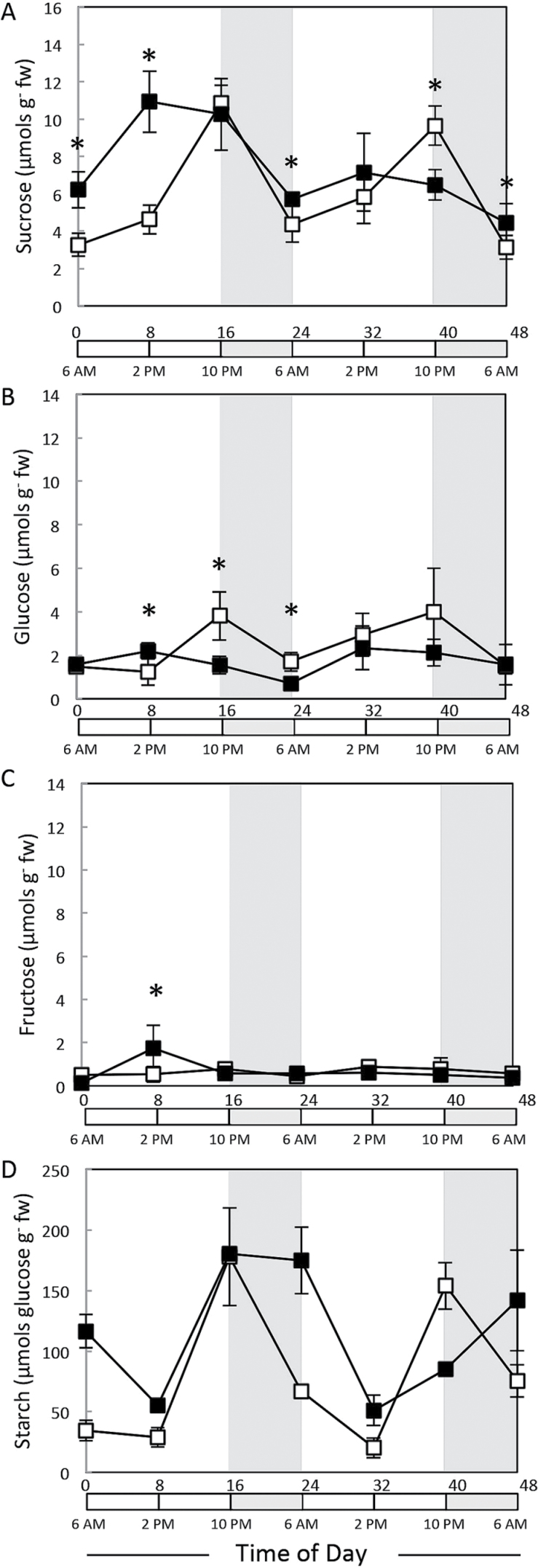
Temporal levels of soluble sugars and starch in maize control or shaded seedlings. Starch and soluble sugars were extracted from the V3 stage control (16h day/8h night, open squares) and shaded (48h, filled squares) plants. Sampling was done every 8h for 48h starting at the end of the night or extended dark period (recovery phase). Sucrose (A, E), glucose (B, F), fructose (C, G), and glucose derived from hydrolysed starch (D, H) was determined by capillary high pressure ion chromatography. Values are presented as means±SE of independent biological replicates (*n*=5).

Under regular diurnal cycling, concentrations of sucrose (Fig. 4A) and starch (Fig. 4D) were lowest in the morning (6 a.m.), rose slightly in the first 8h of light, strongly between 8 and 16h in the light, and decreased overnight. Starch levels were higher than sucrose at dusk, indicating that it was the major transient carbon store in maize. The delay in the onset of starch accumulation resembled that seen in *Arabidopsis* in long photoperiods (Sulpice *et al.*, 2014). Extended starvation stress affected starch and sucrose accumulation, but each in a different way (Fig. 4; filled squares). Unexpectedly, both were higher in leaf 3 at the end of a 48h period of shading than at the end of the 8h night, including a 3-fold higher level of starch. This was in contrast to whole *Arabidopsis* rosettes, where starch and sucrose were very low after 48h of darkness (Usadel *et al.*, 2008). During recovery, sucrose accumulated during the light period, but this increase occurred earlier, by at least 8h (Fig. 4A). This response was attenuated on the second day, indicating a return to a regular diurnal pattern of regulation. During recovery from shading, starch showed a dramatically different response to that in an undisturbed light/dark cycling. Starch decreased during the first 8h in the light and rose to a high level at dusk similar to that in control plants but remained high at the end of the night instead of being degraded (Fig. 4D). The decrease in starch during the first part of the light period coincided with an increase in sucrose levels (compare Fig 4A and D). Glucose and fructose had low levels and a less distinct diurnal pattern, and shading had a minor effect on their levels (Fig. 4B and C).

### T6P response to diurnal cycling and recovery from extended darkness

**Fig. 5.**
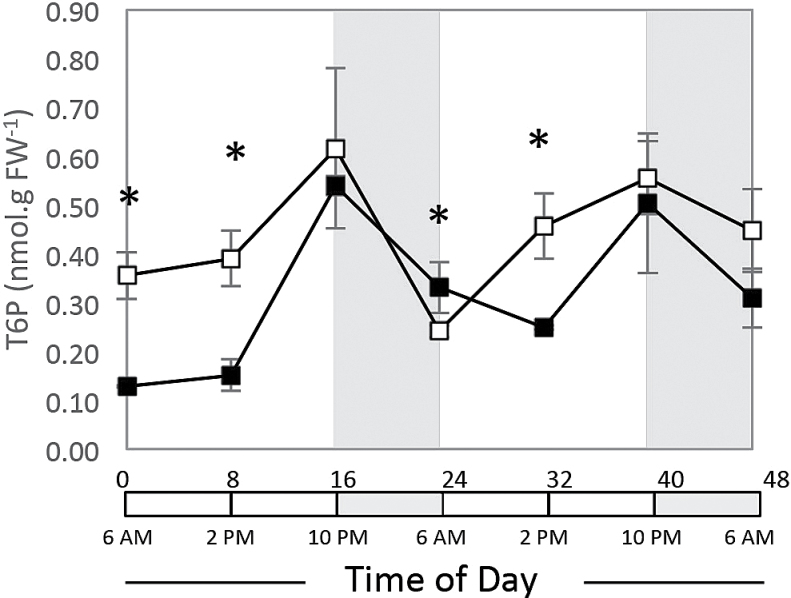
Temporal levels of T6P in maize control or shaded seedlings. Sugars were extracted from the V3 stage control (16h day/8h night, open squares) and shaded (48h, filled squares) plants. Sampling was done every 8h for 48h starting at the end of the night or extended dark period (recovery phase). T6P levels were determined using reversed-phase liquid chromatography, linked to tandem mass spectrometry. Values are presented as means±SE of independent biological replicates (*n*=3).

T6P levels were measured using liquid chromatography coupled with tandem mass spectrometry (Fig. 5, Table 3). Under regular diurnal cycles, T6P levels were low in the morning, went up during the day to reach their maximum in the evening, and then decreased overnight. After 48h of darkness, T6P levels were significantly lower than in control plants in the morning and at noon, and then rose to reach levels like those in the control in the evening. During the night, T6P levels decreased but less than in control plants, and again were lower than in controls at noon the on the second day of recovery, and then rose to levels like those in the control at dusk. Additionally, T6P levels were correlated with sugar and starch levels throughout the diurnal cycle but not after extended darkness (Table 3).

**Table 3.**
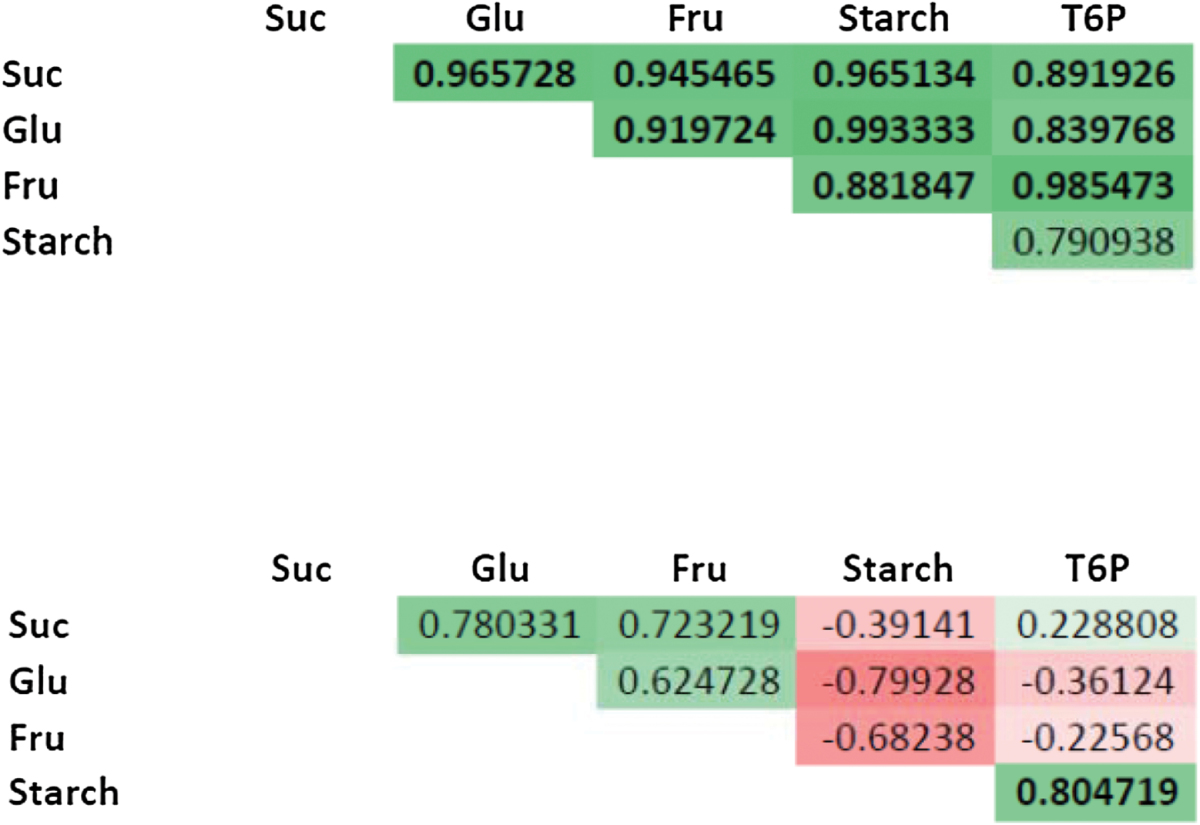
Correlation coefficients between sugars, starch, and T6PCoefficients of correlation were determined over 24h after control (top) and shading (bottom) treatment using a Pearson comparison test (*n*=3–6). Positive and negative correlations are indicated in shades of green and red, respectively.

### TPS/TPP gene expression versus carbohydrate and T6P levels

To help visualize the relationship between TPS/TPP gene expression and sugar levels, we determined the correlation between sugar and transcript levels over the first 24h of treatment using a Pearson test (Table 4). Under control conditions, transcript levels of some class II TPS (*ZmTPSII.4.1*, *-5.3*, and -*5.4*) and SnRK1 upregulated (*ZmAKINβ* and *ZmARG10*) targets negatively correlated with T6P, sucrose, and fructose, and sometimes with glucose and starch. *ZmTPPA.1* behaved similarly and correlated negatively with T6P and fructose. *ZmTPSII.2.1* negatively correlated with sucrose, glucose, and starch, while *ZmTPSII.3.3* correlated negatively with glucose and starch only. Conversely, *ZmTPPA.3* expression correlated positively with T6P, sucrose, and fructose levels. In plants recovering from extended darkness, most class II TPS transcript levels tended to correlate negatively with sucrose and sometimes fructose levels. Conversely, *ZmTPPB.1.3* correlated with sucrose levels. Interestingly both *ZmTPSI.1.1* and *ZmMDH* (malate dehydrogenase) correlated strongly with T6P and starch levels.

**Table 4.**
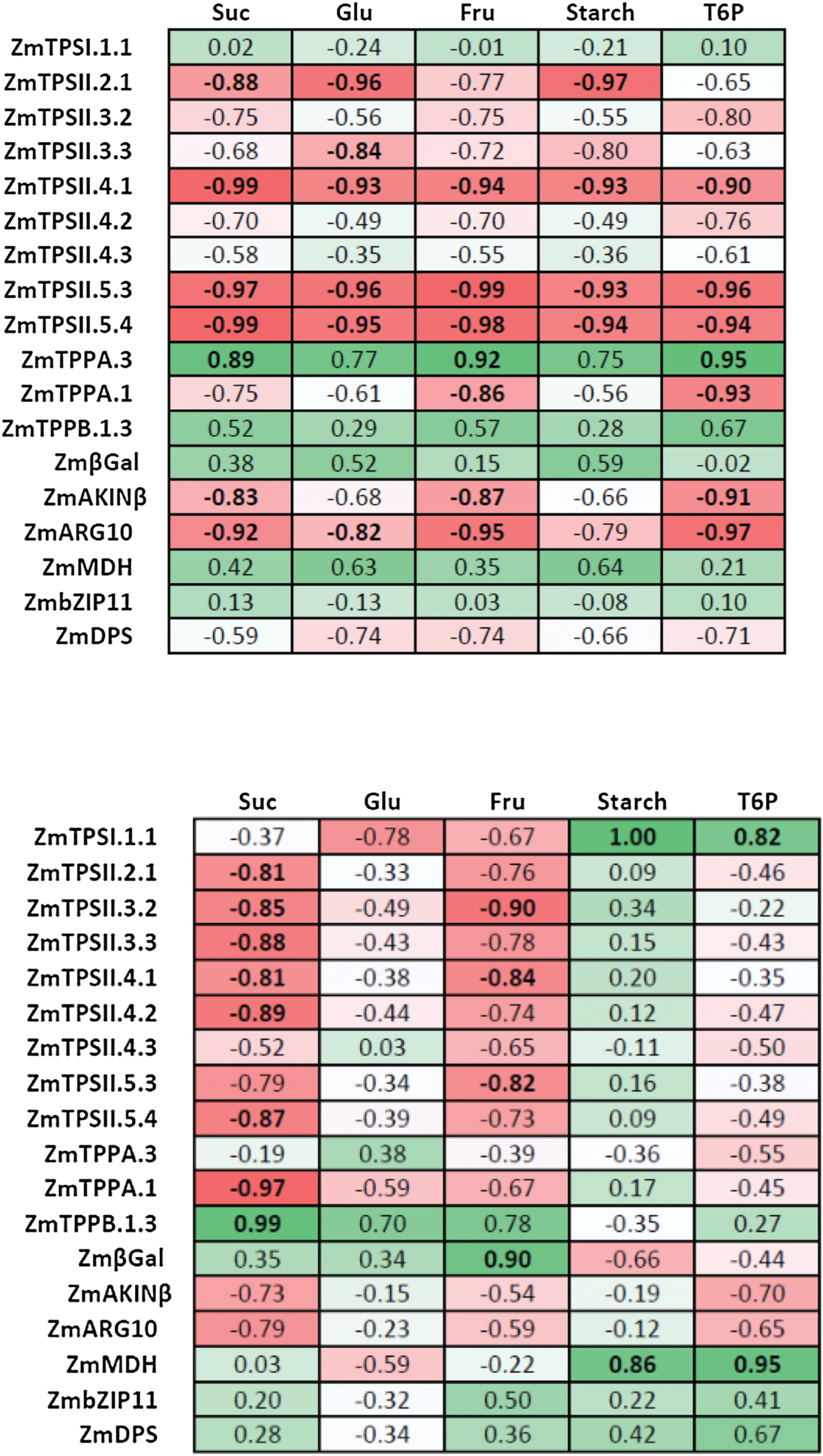
Correlation coefficients between transcripts and sugarsCoefficients of correlation were determined over 24h after control (top) and dark (bottom) treatment using a Pearson comparison test (*n*=3–6). Positive and negative correlations are indicated in shades of green and red, respectively.

## Discussion

Crop productivity depends on the efficient conversion of solar energy into grain and biomass. Many of these efficiencies are realized through the carefully orchestrated metabolic switch between day and night. This large-scale metabolic switch is closely regulated by the circadian clock and through the sensing of intracellular sugar levels (Bläsing *et al.*, 2005). Over the last decade, the trehalose pathway and its intermediate precursor, T6P, have surfaced as key regulators of carbohydrate metabolism, growth, and development in several plant species (reviewed by Paul *et al.*, 2008; Schluepmann *et al.*, 2011; Lunn *et al.*, 2014). Much of the research on the trehalose pathway has used the model C_3_ plant *Arabidopsis*. However, to date little is known of the TPS/TPP gene family and the function of the trehalose pathway and T6P in the regulation of carbon flow and energy status in a C_4_ cereal crop such as maize.

### TPS pathway is conserved in maize

The maize genome encodes families of 14 TPS and 11 TPP genes, while the TRE gene is present in a single copy, which is quite similar to what has been observed in rice, *Arabidopsis*, and poplar (reviewed by Avonce *et al.*, 2006; Lunn, 2007; Li *et al.*, 2008; Paul *et al.*, 2008; Yang *et al.*, 2012). According to evolutionary theories, the duplication process started earlier for TPS genes than for TPP genes. TPS genes have more common ancestors before the split between monocot and dicot species than do TPP genes, which were duplicated more after this event (Fig. 2). The maize class I full-length TPS gene has 16 introns, while most class II TPS genes have two introns (Fig. S1), similar to rice, *Arabidopsis*, and poplar (Yang *et al.*, 2012). Maize TPP genes have a high number of introns (7–10), with the exception of *ZmTPPB.1.2* and -*1.3*, which respectively display five and three introns (Supplementary Fig. S1 at *JXB* online). These results indicate a striking conservation of gene structure across species.

At least one class I TPS gene encodes a catalytic TPS. The maize genome has two class I TPS genes (Fig. 1A, Table 1), with one of them (*ZmTPSI.1.1*, also called *ZmTPS1*) encoding a functional enzyme (Jiang *et al.*, 2010). The other (*ZmTPSI.1.2*) has a truncated TPS domain, which makes it unlikely to be functional. Since its sequence is very similar to *ZmTPSI.1.1*, it may have been duplicated recently, and its function remains unknown. Class I TPS genes also harbour a TPP domain; however, they lack some of the motifs forming the active site of phosphatase proteins belonging to the HAD superfamily (Lunn, 2007; Vandesteene *et al.*, 2010). The maize genome encodes 12 class II TPS genes in four subclades (A2–A5) that include sequences from both monocot and dicots (Fig. 2A). Their structure is similar to *Arabidopsis* class II TPS genes, which have both TPS and TPP domains. As shown in Table 1, they all display both TPS and TPP domains with numerous substitutions in amino acids that are essential for substrate binding and conserved phosphatase motifs (Avonce *et al.*, 2006; Lunn, 2007; Vandesteene *et al.*, 2010). The role for the class II TPS enzymes has been mostly undefined (Chary *et al.*, 2008; Singh *et al.*, 2011); however, the class II TPS genes display remarkable differential spatial and temporal expression patterns in *Arabidopsis* (Ramon *et al.*, 2009). To understand better the possible function for the maize class II TPS proteins, we reviewed the raw data from public genome-wide transcript analyses with attention to class II TPS genes. There were numerous instances where TPS/TPP transcripts showed remarkable spatial and temporal specificity; *ZmTPSII.2.2* in the ovule, *ZmTPSII.3.3*, -*4.2*, -*5.3*, and -*5.4* in the leaf, and *ZmTPSII.5.3* in the endosperm (Davidson *et al.*, 2011; Sekhon *et al.*, 2011). Based on the pattern of expression observed during the diurnal cycle and recovery from darkness, we suggest that the maize class II TPS enzymes play a regulatory role in responding to and/or managing energy resources in seedling leaf tissue, perhaps through its interaction with sugar phosphates.

Close examination of the substrate binding and catalytic domains of the maize class II TPS proteins suggests that this group may not possess catalytic activity (Table 1). R391 has been shown to be required for binding UDPG in the catalytically active TPS1. Substitution of R391 with an aspartate residue in all maize class II TPS proteins may abolish enzymatic activity. This corresponds to the absence of TPS activity observed for most class II TPS in *Arabidopsis* (Vandesteene *et al.*, 2010). Maize class II genes from subclade 5 display more variations in the binding site for UDPG than genes from subclades 2, 3, and 4. Few substitutions are observed, however, in the G6P-binding site (except a minor substitution of T284S). One possible explanation is that the class II TPS proteins have lost their catalytic activity but have retained the binding site for G6P. This may indicate a sensing as opposed to a catalytic function. Such is the case for the plant pathogenic fungi *Magnaporthe grisea TPS1* gene, which has a regulatory function in the pentose pathway required for fungal virulence. This involves G6P binding without formation of T6P, and the association with a regulator protein, TPS3 (Wilson *et al.*, 2007). Since plant and fungal trehalose pathways are somewhat similar (Avonce *et al.*, 2010), a similar process could occur in maize. The existence of high-molecular-weight TPS complexes has already been demonstrated in rice (Zang *et al.*, 2011).

Similar to TPP from other plants, maize TPPs consist of a TPP domain with three conserved phosphatase domains required for activity. Only *ZmTPPB.2.1*, also called RA3, was demonstrated as a functional TPP enzyme in maize where it controls inflorescence branching (Satoh-Nagasawa *et al.*, 2006; Carillo *et al.*, 2013). Genes belonging to subclade A2, A3, or B2 are found only in monocot species, i.e. maize, rice, or sorghum (data not shown), which means that they could have arisen later in evolution or have been lost in dicots. As with TPS genes, several TPP genes show spatial and temporal expression patterns; *ZmTPPA.3* and *ZmTPPB.1.3* in the leaf, *ZmTPPB.2.2* and *-2.3* in anthers and pollen, *ZmTPPA.3* in roots, and *ZmTPPB2.1* in the endosperm, suggesting tissue-specific functions (Davidson *et al.*, 2011; Sekhon *et al.*, 2011).

### TPS/TPP genes show a diurnal pattern of expression

**Fig. 6.**
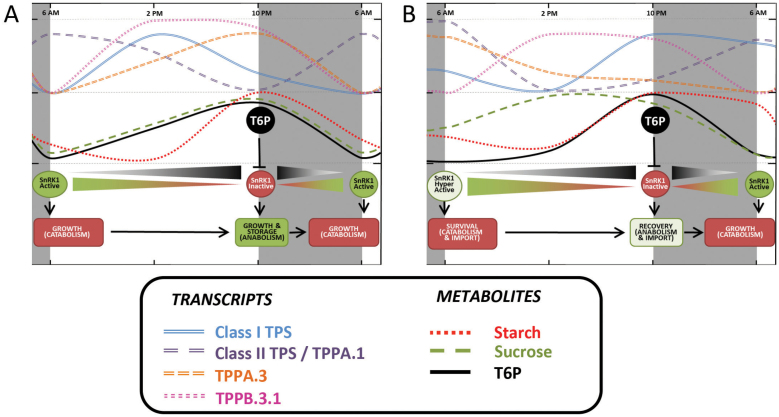
Model depicting a role for the maize trehalose pathway in regulating sugar metabolism and growth under regular day/night cycles and extended darkness in juvenile maize leaves. The model is based on data from the present and previously published work (Baena-González *et al.*, 2007; Zeeman *et al.*, 2007; Zhang *et al.*, 2009; Ghillebert *et al.*, 2011; Wahl *et al.*, 2013) (A) Under regular day/night cycles, as day proceeds, photosynthesis induces the accumulation of sucrose and T6P. Rising levels of T6P gradually promote the inactivation of SnRK1 and the redox activation of AGPase in order to turn on anabolism, growth, and starch accumulation. During the night, starch and sucrose are used as a source of carbon and energy for growth to continue. Their levels, as well as those of T6P, decrease during the night and are low in the morning. Low T6P levels induce: (i) a minimal AGPase (starch synthesis enzyme) activation, preventing starch synthesis; and (ii) a maximal activation of SnRK1 (major energy sensor), enabling growth by partially activating catabolism through starch/sugar consumption. (B) During extended darkness, sucrose and starch immediately available are rapidly used. In order to keep cell stasis, leaf cells slow down metabolism and mobilize alternative carbon sources other leaves, roots, or the attached seed. This generates an accelerated accumulation of remobilized sucrose, negatively correlating with very low T6P levels and a strong activation of SnRK1 at the end of the stress period. SnRK1 activity correlates with the induction of *ZmAKINβ* and *ZmARG10* (positive SnRK1 targets) and all the class II TPS genes, and the repression of *ZmbZIP11* and *ZmDPS* (negative SnRK1 targets) by the end of extended dark period. During recovery, 8h after stress relief, the opposite phenomenon occurs. Photosynthesis is turned on when there remains an abundant supply of imported sucrose, while T6P levels continue to be low because of the perceived stress. Our results indicate that the cell detects intracellularly derived sucrose independently of that which is imported, possibly sensed through hexokinase. Eight hours after returning to the light, T6P levels remain low; however, they increase enough to inactivate SnRK1. Transcript levels change, with a strong repression of *ZmAKINβ* and *ZmARG10* (positive SnRK1 targets) and all the Class II TPS genes, and there is induction of *ZmbZIP11* and *ZmDPS* (negative SnRK1 targets). These SnRK1/T6P-mediated changes result in the switch from growth-from-catabolism to growth-from-anabolism. After 24–48h of recovery, the plant goes back to its regular cycle at the transcriptional level, while metabolites levels are still being affected.

Diurnally expressed genes participate in growth, development, reproduction, and metabolism (Smith *et al.*, 2004; Bläsing *et al.*, 2005; Osuna *et al.*, 2007). So far, the relationship between the trehalose pathway genes and maintenance of energy balance throughout the day/night cycle is not well defined. TPS/TPP gene expression was shown in *Arabidopsis* to be sensitive to sucrose depletion (Thimm *et al.*, 2004; Lunn *et al.*, 2006). In maize, the highest mRNA levels for class II TPS genes were at the end of the regular 8h night period, corresponding to the lowest levels of sucrose, starch, and T6P (Fig. 6A). These results agree with those seen in *Arabidopsis* (Lunn *et al.*, 2006; Wahl *et al.*, 2013). In maize seedlings subjected to a typical diurnal cycle (16h day/8h night), we observed that all class II TPS genes demonstrated a diurnal pattern of gene expression with transcript levels decreasing throughout the day and increasing throughout the night (Fig. 3). Interestingly, T6P levels in the same samples showed a distinct diurnal pattern with levels increasing throughout the day and decreasing throughout the night (Fig. 5). Debast *et al.* (2011) used transgenic potato tubers to produce artificially elevated or reduced T6P levels. They observed that elevated T6P resulted in a reduction of transcripts for two class II TPS genes (*TPS8* and *TPS11*), and repressed T6P levels induced the transcription of these genes. These results in potato corroborate our observation in maize that class II TPS transcripts are inverted with respect to T6P levels throughout the diurnal cycle.

Circadian clock-regulated genes participate in a large number of physiological processes, preparing the plant for the rhythmic change in its environment. In *Arabidopsis*, as much as 30% of the expressed transcripts cycle every 24h under constant light and temperature (Covington *et al.*, 2008). The rhythmic control of circadian-regulated genes continues after cycling environmental cues, i.e. light and temperature, have been removed (Doherty and Kay, 2010; Khan *et al.*, 2010). Kahn *et al.* (2010) set the circadian clock by exposing maize seedlings to 12h light/12h dark, and then switched to continuous light for 48h to identify those genes regulated by the circadian clock. They identified >1300 transcripts that maintained a circadian rhythm even after being switched to continuous light. Here, we identified four TPS genes that were among the data they collected; *ZmTPSI.1.1* and *ZmTPSII.5.3*, -*2.1*, and -*3.2*. For these four TPS genes, transcript levels drift along with complete loss of circadian cycling. At least for these four TPS genes, it can be concluded that gene expression is not regulated by the circadian clock but rather by the energy status of the cell (Gibon *et al.*, 2004).

All of the maize TPS and TPP genes examined in this work showed some degree of diurnal cycling and, based on the results of Khan *et al.* (2010), are regulated by energy status as opposed to an internal clock. This provides further evidence that regulation of the trehalose pathway is tightly linked to sugar levels and plays an important role in maintaining sensing and energy stasis. Usadel *et al.* (2008) observed transcriptome changes in vegetative *Arabidopsis* rosettes throughout the diurnal cycle and after 4h of extended night. Examination of their data revealed that, as with maize, under regular diurnal cycling all class II TPS genes showed diurnal cycling with highest expression at the end of the night period, with the exception of *AtTPS5*, which has no homologue in maize (Figs 2 and S3B).

In contrast to the class II genes, *ZmTPSI.1.1* is expressed at its lowest levels during the night and rises during the day, which is out of phase with sucrose and T6P levels, again in agreement with that seen with all class I genes in *Arabidopsis* (Fig. S3A) (Usadel *et al.*, 2008). Low transcript levels at the end of night and at end of the extended darkness indicate that *ZmTPSI.1.1* is not likely to be under the control of SnRK1 as with the class II genes (Fig. 6). *ZmTPSI.1.1* is perhaps regulated at the post-translational level through interaction with specific kinases, e.g. SnRK1, phosphatases, or class II TPS proteins (Glinski and Weckwerth, 2005; Harthill *et al.*, 2006). We also observed that gene expression patterns for the three predominant maize TPP genes, *ZmTPPA.3*, *ZmTPPB.1.3*, and *ZmTPPA.1*, was varied and showed unique patterns of expression. *ZmTPPA.3* and *ZmTPPB.1.3* had the lowest expression at the end of the night period, in contrast to what was observed for class II TPS transcripts, and is consistent with their role in the dephosphorylation of T6P (Lunn *et al.*, 2006). *ZmTPPA.1* followed an expression pattern similar to the class II TPS genes, with the highest expression at the end of the dark period. This variable pattern for TPP genes is the same as seen in *Arabidopsis* (Fig. S3C) (Usadel *et al.*, 2008).

### Effect of extended darkness on energy status and TPS/TPP gene expression

It has been shown previously that reducing light by as little as 30% can have significant impact on grain production and total biomass yield in maize (Earley *et al.*, 1966). Setter *et al.* (2001) imposed 5 d of shade stress on flowering maize plants. They observed a 66% reduction in kernel dry matter production, along with a 20–50% reduction in floret carbohydrates. Here, we also observed that extended darkness (48h) had a significant effect on starch and sucrose levels in maize seedlings (Fig. 4). Surprisingly, 48h of darkness resulted in higher levels of sucrose and starch, suggesting a slowing of carbon usage for growth including cell expansion (Fig. 2) and the mobilization of carbohydrate reserves stored in other leaves, roots, or the attached seed. During a regular light/dark cycle, the plant draws on cellular reserves of sugar and starch to fuel metabolism and growth. Our results indicate that, during prolonged darkness, the plant enters a metabolic stasis in order to survive.

Perhaps most surprising was the observation that when plants were returned to a light/dark cycle after extended darkness, T6P levels no longer followed the same time course as sucrose and hexose sugars. When *Arabidopsis* plants were subjected to extended nights (Usadel *et al.*, 2009), during leaf senescence (Wingler *et al.*, 2012), were starved for carbon (Yadav *et al.*, 2014), or in the maize seedling leaf under a regular diurnal cycle (Fig. 6), T6P levels always followed the same time course as sucrose and hexose sugars. We suggest that, after a period of darkness, leaf 3 does not sense sugars imported from other parts of the plant in the same manner as cellular-derived sugar sources. Certainly the cell does not appear to sense imported sugars through T6P or SnRK1. The difference may lie in the mechanism of sucrose degradation i.e., invertase, sucrose synthase, and the products of these reactions, i.e. glucose, fructose, UDPG, or modifications in the sucrose sensing pathways.

Schussler and Westgate (1995) suggested that it is the flux of carbohydrates into the developing ovary as opposed to sugar concentration per se that determines kernel set. A possible mechanism for sugar sensing could be through the rapidly turning over pool of intermediates such as UDPG and G6P (Setter *et al.*, 2001). These are also substrates for TPS, or are capable of binding to a catalytically inactive TPS protein. Usadel *et al.* (2009) observed the effect of extending the night by an additional 6h for maize seedlings. Using the maize 18K Affymetrix chip, they found that extending the dark (similar to short days for *Arabidopsis*) resulted in a 2- to 4-fold increase in transcripts for several class II TPS genes (*ZmTPSII.4.1*, -*5.2*, and -*5.3*), suggesting that class II TPS enzymes participate in maintaining the survival state through its sensing of sugars. We observed that all class II TPS transcripts that were typically at their highest level at 6 a.m. were several orders of magnitude higher after 48h of darkness, and dropped rapidly as sucrose levels rose in the light (Fig. 3). These results suggest an important role for the maize class II TPS enzymes in prolonging survival and in recovering from extended darkness. As before, *Arabidopsis* class II TPS genes are induced by extended night, with the exception of *AtTPS5* (Usadel *et al.*, 2008). Osuna *et al.* (2007) starved *Arabidopsis* seedlings grown in liquid culture under low light by withholding sucrose for 48h. They observed a rapid (30min) sucrose-dependent alteration in transcripts for more than 1000 genes, including a decrease in *AtTPS8*, *AtTPS9*, *AtTPS10*, and *AtTPS11*. One possible explanation for the pattern seen in Fig. 4A is that class II genes are expressed when sucrose levels are low and SnRK1 is active. This result is consistent with the transcriptional co-regulation of various TPS genes by energy related stresses (sucrose starvation, darkness, etc.), and the SnRK1 catalytic subunit KIN10 in *Arabidopsis* (Baena-González, 2007; Ghillebert *et al.*, 2011).

A very different response was observed for the catalytically active *ZmTPSI.1.1* in that extended darkness resulted in repression of the transcript at a time it normally peaks during the diurnal cycle (2 p.m.). Indeed we found that, in plants recovering from extended darkness, the *ZmTPSI.1.1* transcript closely mimicked starch levels (*R* ^2^=99%). The relationship between the *ZmTPSI.1.1* transcript and starch levels after extended darkness indicates a metabolic shift from short-term sugar depletion (8h) to long-term absence of photosynthate (48h) with a possible role for starch in the formation, hydrolysis, and/or sensing to regulate *ZmTPSI.1.1* levels. This hypothesis could be supported by the results of Scialdone *et al.* (2013), indicating that *Arabidopsis* plants sense both starch and day length in order to regulate starch degradation rate. Such a phenomenon could be involved in regulation of target metabolic genes to enable the plant to adjust its environment according to its internal resources.

### Role of TPS, TPP, and T6P in sugar sensing and maintenance of energy status

Based on literature from *Arabidopsis* and potato, as well as our results, we present a model for the regulation of energy balance throughout the diurnal cycle and the recovery from extended darkness that incorporates the trehalose pathway genes T6P and SnRK1 (Fig. 6). During a typical night period, starch is consumed to maintain growth and cellular metabolism. Starch breakdown provides less sucrose than carbon fixation in the light, and thus sucrose levels fall as reflected by a decrease in T6P levels, with a peak at dusk and a minimum at dawn (Lunn *et al.*, 2006; Wahl *et al.*, 2013). This occurs coincidentally with an observed increase in transcription of SnRK1 target (inducible) genes (Usadel *et al.*, 2008) (Supplementary Fig. S3D, E). AGPase is then inactivated by changes in allosteric regulators and by light- and sucrose-dependent post-translational redox modification, while starch degradation is stimulated (Tiessen *et al.*, 2003; Gibon *et al.*, 2004; Kolbe *et al.*, 2005; Lunn *et al.*, 2006). SnRK1 activity is also induced during the night as the plant enters sink mode (Baena-González *et al.*, 2007), correlated with the transcription of class II TPS genes in maize, such that their peak expression is at the end of the night period, and this may result in SnRK1-mediated phosphorylation of some TPS1 (Glinski and Weckwerth, 2005), resulting in feedback regulation of the trehalose pathway. Upon re-illumination, sucrose and starch accumulate and T6P levels rise (Wahl *et al.*, 2013), inhibiting SnRK1 (Zhang *et al.*, 2009). This is accompanied by the activation of AGPase, repression of starch degradation, upregulation of *ZmTPSI.1.1* gene expression, and downregulation of class II TPS transcription. These transcriptional and metabolic changes are consistent with cell growth, with its optimum at the end of the day.

Interestingly, in maize seedling leaf tissue, each of the eight class II TPS genes showed the same pattern of transcript induction during the night, although these genes show quite varied expression throughout development and in response to environmental stimuli (Covington *et al.*, 2008; Wahl *et al.*, 2013). The only maize TPS gene known to have catalytic function, *ZmTPSI.1.1*, was not induced after extended darkness; however, was induced during the afternoon. Extended darkness resulted in even lower transcript levels for *ZmTPSI.1.1*, an indication that transcriptional regulation of this TPS gene is critical during normal growth and not while the plant is subjected to prolonged darkness. One can infer from this that: (i) *ZmTPSI.1.1* expression does not require SnRK1 to be active; or (ii) its transcriptional regulation is not important in the production of stress-induced T6P. Our attention now turns to the class II TPS genes in maize in regard to their role in sugar metabolism and during the recovery from extended darkness.

## Conclusions

The maize family of trehalose biosynthetic enzymes offers a fascinating system for the characterization of energy management with respect to sucrose and starch, and how it contributes to crop productivity and stress tolerance. Regarding the present results, recovery from extended darkness probably involved the participation of class II TPS proteins. It is of great interest to determine their role in this process, whether they can function catalytically alone or as regulatory elements of a high-molecular-weight complex, or if they act as signalling molecules or transcription factors. Further investigation into protein–protein interactions will validate this hypothesis. The use of mutants and transgenic plants will facilitate our understanding of how each TPS and TPP enzyme contributes to what is undoubtedly a complex regulatory apparatus. We also observed that extended darkness disrupted the connection between sucrose and T6P, suggesting multiple sucrose sensing pathways operating simultaneously.

## Supplementary data

Supplementary data are available at *JXB* online.

Supplementary Methods S1. Methods for sugars and starch analysis.

Supplementary Method S2. Formulae for sugar and starch (glucose) analysis.

Supplementary Table S1. Gene names and accession numbers for maize, rice, *Arabidopsis*, and poplar TPS and TPP.

Supplementary Table S2. Sequence of primers used for quantitative PCR.

Supplementary Fig. S1. Gene structures with introns for maize TPS I and II genes.

Supplementary Fig. S2. Predicted enzymatic domains for maize TPS and TPP genes.

Supplementary Fig. S3. *Arabidopsis* gene expression

Supplementary Data

## Abbreviations:

G6P: glucose-6-phosphate
HXK: hexokinase
RT: reverse transcription
SE: standard error
T6P: trehalose-6-phosphate
TPP: trehalose-6- phosphate phosphatase
TPS: trehalose-6-phosphate synthase
TRE: trehalose
UDGP: uridine diphosphate glucose.
