# Supplementary material for: The trehalose pathway in maize: conservation and gene regulation in response to the diurnal cycle and extended darkness
Source: J Exp Bot. 2014 Sep 30;65(20):5959–73. doi: 10.1093/jxb/eru335 (PMC4203130; doi:10.1093/jxb/eru335)
Supplement: Supplementary Data [file supp_65_20_5959__index.html]

The trehalose pathway in maize: conservation and gene regulation in response to the diurnal cycle and extended darkness — The trehalose pathway in maize: conservation and gene regulation in response to the diurnal cycle and extended darkness — Supplementary Data 

# The trehalose pathway in maize: conservation and gene regulation in response to the diurnal cycle and extended darkness

## Supplementary Data

Data files

**Files in this Data Supplement:**

- Supplementary Data - Supplementary Data
- Supplementary Data - Supplementary Data
