# Supplementary material for: The trehalose pathway in maize: conservation and gene regulation in response to the diurnal cycle and extended darkness
Source: J Exp Bot. 2014 Sep 30;65(20):5959–73. doi: 10.1093/jxb/eru335 (PMC4203130; doi:10.1093/jxb/eru335)
Supplement: Supplementary Data [file supp_eru335_jexbot127621_file002.pdf]

SUPPLEMENTARY DATA

Supplemental Methods S1. Methods for sugars and starch analysis.
Supplemental Method S2. Formulae for sugar and starch (glucose) analysis.
Supplemental Table T1. Gene names and accession numbers for maize, rice, Arabidopsis, and poplar TPS and TPP.
Supplemental Table T2. Sequence of primers used for qPCR.

Carbohydrate Metabolite Analysis

Frozen tissues (20-100 mg) were weighed and ground for 30-60 s while frozen using a Tissue Lyser II (Qiagen). Sugars (sucrose, hexoses and starch) were then extracted following a method adapted from Lunn et al. 2006. Tissues were resuspended in 500 µL of ice cold CHCl3/CH3OH (3:7, v/v). 250 nmols of lactose was added as an internal standard to calculate recovery. Soluble sugars were extracted for 2 h at -10°C with shaking in an orbital mixer (Model 5C25, Cole Parmer) for 5 min every 15 min interval, followed by two extractions with 400 and 200 µL of water. Samples were then vortexed, incubated for 5 min at 4°C, centrifuged for 5 min at 10,000g and 200 µL of the upper aqueous phase was collected and dried using a centrifugal vacuum. The residue was resuspended in 250 µL of water and filtered by centrifugation for 2 h at 2250g using a MultiScreen® Ultracel-10 filter plates (Millipore) with samples covered with mineral oil to prevent evaporation. The filtrate was used directly for sugars analysis as described below. Starch was extracted from the pellet generated while extracting soluble sugars. The remaining upper phase was removed and the pellet was washed with 1 mL ice cold CH3OH. The supernatant was removed and the pellet was dried using a centrifugal vacuum. The dried material was resuspended with 200 µL 0.5 M NaOH and incubated for 1 h at 60°C with shaking to dissolve the starch. The solution was neutralized with 200 µL of 0.5 M HCl, and the starch digested in 1 U amyloglucosidase (Roche Diagnostics), 600 µL 0.2 M NaOAc (pH 4.5) and 1 µmol lactose as internal standard for 12 to 24 h at 30°C with shaking. The reaction was stopped by boiling samples for 2 min. Samples were then centrifuged to remove debris and the supernatant was transferred to a new microcentrifuge tube. 250 µL of digested starch was filtered as described previously for soluble sugars. Samples were diluted 1:100 in filtered ultrapure water for soluble sugars and 1:10 for hydrolyzed starch.

Carbohydrate Metabolite Analysis

Frozen tissues (20-100 mg) were weighed and ground for 30-60 s while frozen using a Tissue Lyser II (Qiagen). Sugars (sucrose, hexoses and starch) were then extracted following a method adapted from Lunn et al. 2006. Tissues were resuspended in 500 µL of ice cold CHCl3/CH3OH (3:7, v/v). 250 nmols of lactose was added as an internal standard to calculate recovery. Soluble sugars were extracted for 2 h at -10°C with shaking in an orbital mixer (Model 5C25, Cole Parmer) for 5 min every 15 min interval, followed by two extractions with 400 and 200 µL of water. Samples were then vortexed, incubated for 5 min at 4°C, centrifuged for 5 min at 10,000g and 200 µL of the upper aqueous phase was collected and dried using a centrifugal vacuum. The residue was resuspended in 250 µL of water and filtered by centrifugation for 2 h at 2250g using a MultiScreen® Ultracel-10 filter plates (Millipore) with samples covered with mineral oil to prevent evaporation. The filtrate was used directly for sugars analysis as described below. Starch was extracted from the pellet generated while extracting soluble sugars. The remaining upper phase was removed and the pellet was washed with 1 mL ice cold CH3OH. The supernatant was removed and the pellet was dried using a centrifugal vacuum. The dried material was resuspended with 200 µL 0.5 M NaOH and incubated for 1 h at 60°C with shaking to dissolve the starch. The solution was neutralized with 200 µL of 0.5 M HCl, and the starch digested in 1 U amyloglucosidase (Roche Diagnostics), 600 µL 0.2 M NaOAc (pH 4.5) and 1 µmol lactose as internal standard for 12 to 24 h at 30°C with shaking. The reaction was stopped by boiling samples for 2 min. Samples were then centrifuged to remove debris and the supernatant was transferred to a new microcentrifuge tube. 250 µL of digested starch was filtered as described previously for soluble sugars. Samples were diluted 1:100 in filtered ultrapure water for soluble sugars and 1:10 for hydrolyzed starch.

Sugar peaks were identified in comparison with known sugars anc following formulas:

Soluble sugars:

(1)

$$Initial\ sugar\ quantity\ [\mu mol\ g^{-1}\ of\ FW] = \frac{FSC\ \times FV\ \times L}{}$$

Where:

Final sugar concentration:

FSC = Sugar peak area [nC] / Slope of standard curve for this sugar [nC = f(sugar

Final volume of resuspension:

FV = $2.5x10^{-4}$ *L*

Lost correction factor:

LCF = Standard lactose peak area for a final concentration of 100 µM / Actual lact

**A final concentration of 100 µM lactose would have been expected if adding 25 µL internal standard and not diluting samples before running. Since Dionex optimal sen concentrations from 1 to 100 µM, and that kernel samples contains high quantity of 1/100 and increase initial lactose to sugar ratio by 100.*

Lactose-Sugar Ratio:

L / S Ratio = 10

(2) Glucose from starch digestion:

$$Initial\ glucose\ quantity\ [\mu mol\ g^{-1}\ of\ FW] = \frac{FGC\ \times FV\ \times}{}$$

Where:

Final glucose concentration:

FGC = Glucose peak area [nC] / Slope of standard curve for glucose [nC = f(glucos

Final volume of resuspension:

FV = 10^{-3} *L*

Lactose-Glucose Ratio:

L / G Ratio = 10

d data analysed using the

$$\frac{LCF \times L / S \ Ratio}{FW}$$

concentration)]

*ose peak area **
of 1 mM of lactose as an
sitivity applies to sugar
sugars, we had to dilute them to

$$\frac{LCF \times L / G \ Ratio}{FW}$$

se concentration)]

Supplemental table S1: TPS and TPP genes names and accession numbers in maize, rice, Arabidopsis and poplar

Specie	Gene name	Accesion number in PLAZA	Accesion number in other databases	Database
**Maize**	ZmTPSI.1.1_tps1	ZM08G19270	GRMZM2G068943_T01	www.maizesequence.org
	ZmTPSI.1.2	ZM06G28170	GRMZM2G001304_T01	
	ZmTPSII.2.1	ZM07G13460	GRMZM2G019183_T02	
	ZmTPSII.2.2	ZM01G08410	GRMZM2G099860_T01	
	ZmTPSII.3.1	ZM03G31900	GRMZM2G304274_T01	
	ZmTPSII.3.2	ZM03G31920	GRMZM2G123277_T01	
	ZmTPSII.3.3	ZM08G19270	GRMZM2G118462_T01	
	ZmTPSII.4.1	ZM02G30010	GRMZM2G527891_T01	
	ZmTPSII.4.2	ZM01G39130	GRMZM2G008226_T01	
	ZmTPSII.4.3	ZM04G13350	GRMZM2G366659_T01	
	ZmTPSII.5.1	ZM04G11490	GRMZM2G007736_T02	
	ZmTPSII.5.2	ZM01G37720	GRMZM2G079928_T01	
	ZmTPSII.5.3	ZM04G26220	GRMZM2G312521_T01	
	ZmTPSII.5.4	ZM05G42890	GRMZM2G122231_T01	
	ZmTPPA.1	ZM09G18330	GRMZM2G178546_T01	
	ZmTPPA.3	ZM05G35700	GRMZM2G112830_T01	
	ZmTPPB.1.1	ZM01G39020	GRMZM2G347280_T01	
	ZmTPPB.1.2	ZM02G29850	GRMZM2G140078_T01	
	ZmTPPB.1.3	ZM07G12490	GRMZM2G174396_T01	
	ZmTPPB.1.4	ZM05G40760	GRMZM2G055150_T01	
	ZmTPPB.1.5	ZM04G27640	GRMZM2G151044_T01	
	ZmTPPB.1.6	ZM09G00990	GRMZM2G080354_T01	
	ZmTPPB.2.1_ramora3	ZM07G27620	GRMZM2G014729_T01	
	ZmTPPB.2.2	ZM02G39290	GRMZM2G117564_T01	
	ZmTPPB.2.3	ZM07G27610	GRMZM5G840145_T01	
**Rice**	OsTPS1	OS05G44210	HM050424	Genebank
	OsTPS2	OS01G54560	HM050425	
	OsTPS3	OS01G53000	HM050426	
	OsTPS4	OS03G12360	HM050427	
	OsTPS5	OS02G54820	HM050428	
	OsTPS6	OS05G44100	HM050434	
	OsTPS7	OS08G31980	HM050429	
	OsTPS8	OS08G34580	HM050430	
	OsTPS9	OS09G25890	HM050431	
	OsTPS10	OS09G23350	HM050432	
	OsTPS11	OS09G20990	HM050433	
	OsTPP1	OS02G44230	AB120515	

	OsTPP2	OS10G40550	AB277360
	OsTPP3	OS07G43160	NM_001066861
	OsTPP4	OS02G51680	NM_001054678
	OsTPP5	OS04G46760	TPP5_ORYSJ
	OsTPP6	OS08G31630	TPP6_ORYSJ
	OsTPP7	OS09G20390	BAD25928
	OsTPP8	OS06G11840	BAD37685
	OsTPP9	OS03G26910	AAT78804
	OsTPP10	OS07G30160	NP_001059655
	OsTPP11	OS02G44235	*none*
	OsTPP12	OS10G40555	OsI_34594
	OsTPP13	OS12G09060	LOC_Os12g09060
**Arabidops**	AtTPS1	AT1G78580	
	AtTPS2	AT1G16980	
	AtTPS3	AT1G17000	
	AtTPS4	AT4G27550	
	AtTPS5	AT4G17770	
	AtTPS6	AT1G68020	
	AtTPS7	AT1G06410	
	AtTPS8	AT1G70290	
	AtTPS9	AT1G23870	
	AtTPS10	AT1G60140	
	AtTPS11	AT2G18700	
	AtTPPA	AT5G51460	
	AtTPPB	AT1G78090	
	AtTPPC	AT1G22210	
	AtTPPD	AT1G35910	
	AtTPPE	AT2G22190	
	AtTPPF	AT4G12430	
	AtTPPG	AT4G22590	
	AtTPPH	AT4G39770	
	AtTPPI	AT5G10100	
	AtTPPJ	AT5G65140	
**Poplar**	PtTPS1	PT11G09730	
	PtTPS2	PT01G38460	
	PtTPS3	PT03G08380	
	PtTPS4	PT08G13400	
	PtTPS5	PT01G02000	
	PtTPS6	PT10G10370	
	PtTPS7	PT11G06720	
	PtTPS8	PT04G05810	
	PtTPS9	PT12G07420	
	PtTPS10	PT15G07580	
	PtTPS11	PT06G17480	

PtTPS12	PT18G09730
PtTPS14	PT04G06150
PtTPPA.1.1	PT12G13940
PtTPPA.1.2	PT15G13860
PtTPPA.1.3	PT01G00080
PtTPPA.1.4	PT03G10240
PtTPPB.1.1	PT05G15960
PtTPPB.1.2	PT02G09320
PtTPPB.1.3	PT05G07700
PtTPPB.1.4	PT07G05480

Supplemental table S2: Maize TPS, TPP, SnRK1 targets and reference genes RT-qPCR primers sequence, product size and efficiency

Gene	Gene accession number	Primers sequence	Product size (bp)	Efficiency
ZmTPSI.1.1_tps1	GRMZM2G068943	ACAGAGCTACACCCGTAGCTAGTCA<br>TCCTTTATCCTTTCCCATTTGCTA	107	1.81
ZmTPSII.2.1	GRMZM2G019183	AGCTACGGTCAGTCCTCAACC<br>GAAGATATCCATGTCATCAACACCA	116	1.84
ZmTPSII.3.2	GRMZM2G123277	GCATCGGCGATGATAGGTCC<br>AATCAGATTCCAGTTCAGCTCCAGT	210	1.99
ZmTPSII.3.3	GRMZM2G118462	TTTGAAAATATTGCTGATATCATTGG<br>GATTGTTCGTCACCAATATCAAGTG	233	2
ZmTPSII.4.1	GRMZM2G527891	CTCCAAGCGCTGAACTTATCTCTAC<br>GCTTCCATTCAGAATAAATACCTGAGA	216	1.92
ZmTPSII.4.2	GRMZM2G008226	ATTTCTTGATTACGATGGCACACTT<br>CCGCTAGACCAAGCTTCTCACAC	185	1.97
ZmTPSII.4.3	GRMZM2G366659	TGTGAAGTGTGGCCATTATATCGTA<br>CGTTGTTGTTGGCCAGTGCT	98	1.93
ZmTPSII.5.3	GRMZM2G312521	ATGTTTGGACCACCTTTGTATATGG<br>CGGAGTGAATGAATCAACTTCTCTT	100	1.71
ZmTPSII.5.4	GRMZM2G122231	CCATGGGATACCTCCGGG<br>CTCTCCTTGTGCTCGATGTAGGAG	113	2
ZmTPPA.1	GRMZM2G178546	GGCGGAAGATGACTATAAAAAGGTT<br>AGCGATTCAAGTAAAAACTCCACAG	156	2.05
ZmTPPA.3	GRMZM2G112830	GTCACCTGTCATCACCGATCC<br>ATTGACAAGGACCTCCTCGATTTTA	157	1.95
ZmTPPB.1.3	GRMZM2G174396	GCCAAGGCCTCCTCTTCTTCT<br>CAGAACCTGTTGTTCTCCACCTTG	158	2.11
ZmβGal	ZM03G38190	GGATTGCCAGGGTTTACAGGA<br>CTAACCACTTCTTCCATGCAAGTCT	100	2.07
ZmAKINβ	ZM09G22070	GTTTGCTGTTACAGAGAGCCAAGG<br>TTTCATTCCTGGGATGGGATG	95	2.12
ZmARG10 *	ZM10G26580	CAACCACGAGACTTTGCTTCTAAAC<br>CTAGGCAGGAAAAGTGAAAAAGGAT	100	2.03
ZmMDH	ZM04G14160	ATGGTTGCTGTTTGTTTCTAATTG<br>GTGTAAATAAGGCCTGGTTCAGAAA	90	2.11
ZmbZIP11	ZM04G40980	GTGTACTGTGTCACTCCACTCCAAC<br>ATTGTATGGTGCACTTCCTTTGTTT	92	1.93
ZmDPS *	ZM06G24060	CGGCTCAGGACTCCCATTT<br>GCCGAGGCTTGAGATTGATAG	87	2.05
ZmEF1α-1	GRMZM2G153541 (at1g07920)	AATCTCTGGTTTTGAAGGTGACAAC<br>CAAAAGTAACAACCATACCAGGCTTA	230	1.9
ZmEF1α-2	GRMZM2G343543 (at5g60390)_1	AAGACTTATCCGAACATCTGGTGAG<br>AGATTTAAGCGCAAGAGAAATTTGA	163	2.05
ZmEF1α-3	GRMZM2G112158 (at1g07940)	ACAGATGAAGTTGCTAAATCCAAGC<br>GTACTCATTTCAGGAAGCAGGTCAT	170	2.05
ZmPP2AA2-2	GRMZM2G122135 (at3g25800)_3	TACCTGTAATTTGTTGGGCCTTTTA<br>TACGTGTTGTGTCCTGCTCAATTAT	151	1.97
ZmCACS	GRMZM2G331032 (at5g46630)	CTGGGATTAAATGACAAGATTGGAC<br>ACCCTCTGTGATTCGATACTTCATC	201	2.14
ZmCDC27	GRMZM2G392710 (at2g20000)	GATGGAAGATCCTTTGAGCAAGATA<br>TTTCTGTAGACTTCCAGTGCTTCCT	164	2
