# Supplementary material for: The trehalose pathway in maize: conservation and gene regulation in response to the diurnal cycle and extended darkness
Source: J Exp Bot. 2014 Sep 30;65(20):5959–73. doi: 10.1093/jxb/eru335 (PMC4203130; doi:10.1093/jxb/eru335)
Supplement: Supplementary Data [file supp_eru335_jexbot127621_file001.pdf]

Supplemental Figures S1, S2, and S3

TITLE: The Trehalose Pathway in Maize: Conservation and Gene Regulation in Response to Diurnal Cycle and Extended Darkness

AUTHORS: Clémence Henry, Samuel W. Bledsoe, Allison Siekman, Alec Kollman, Brian M. Waters, Regina Feil, Mark Stitt and L. Mark Lagrimini

Supplemental Figure Legends

Figure S1. Gene structures with introns for maize TPS I and II genes (A) and TPP genes (B). Graphical representation of maize TPS genes showing exons (blue), introns (gray), and non-coding sequences (green).

Figure S2. Predicted enzymatic domains for maize TPS (A) and TPP (B) genes. Conserved residues for substrate binding are highlighted in red.

Figure S3. Expression of Arabidopsis (A) class I TPS genes, (B) class II TPS genes, (C) TPP genes, (D) SNRK1 (inducible) targets, (E) SnRK1 (repressible) targets in mature leaf tissue throughout the diurnal cycle and after 4 h extended night.

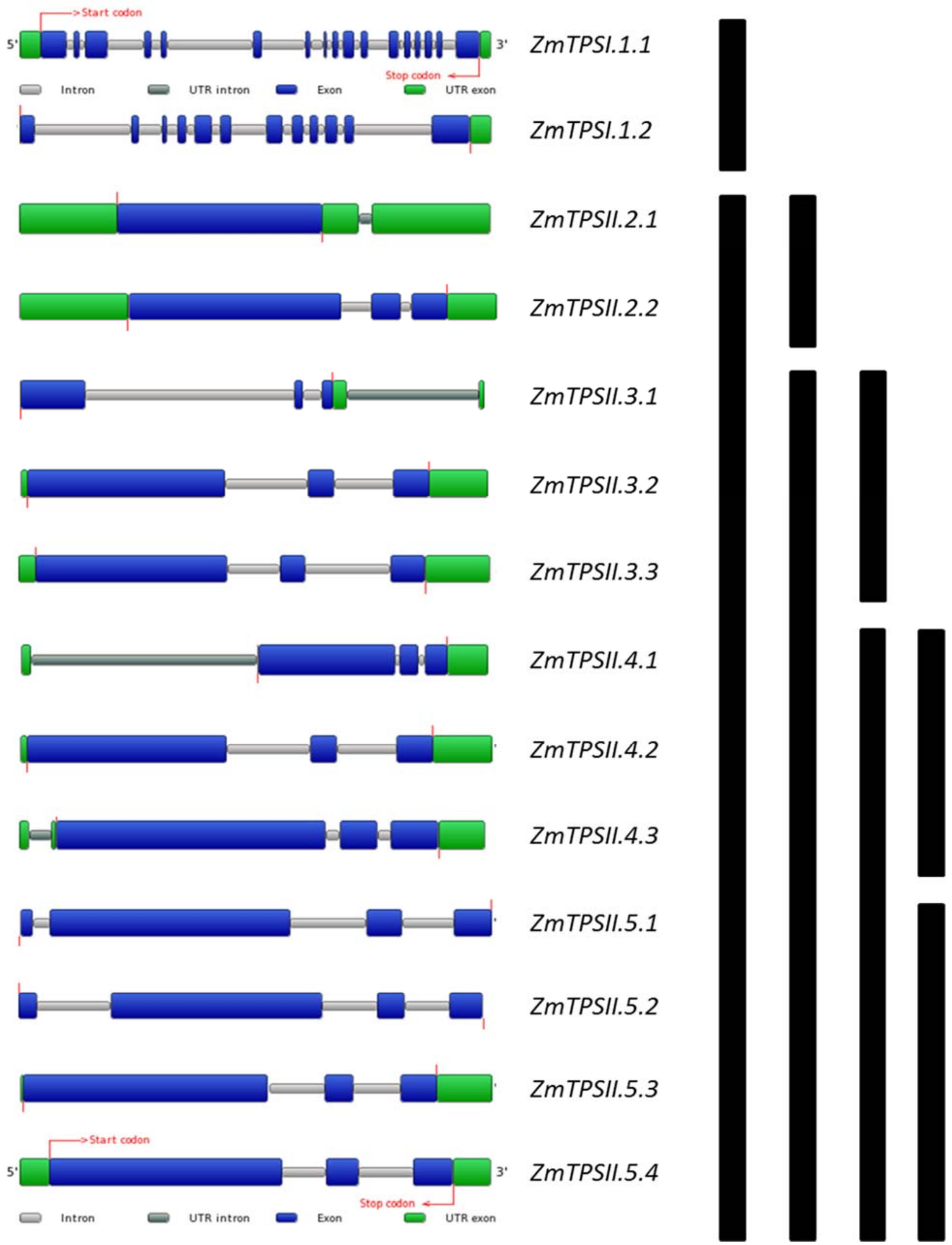

Supplemental Figure S1A: Gene structures with introns for maize TPS class I and class II genes.

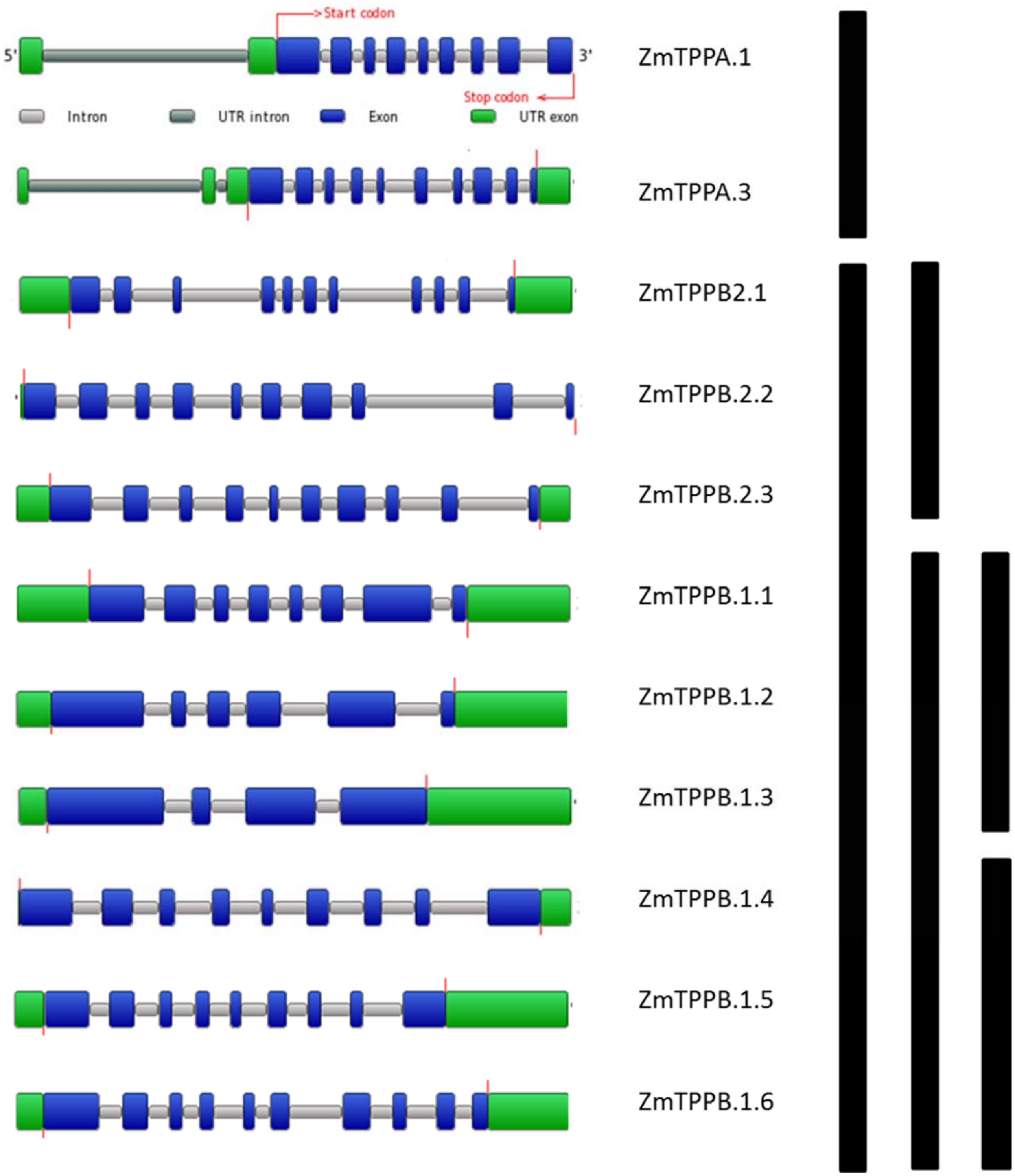

Supplemental Figure S1B: Gene structures with introns for maize TPP class A and class B genes.

Supplemental Figure S2A: Predicted enzymatic domains for TPS proteins.

TPS predicted domain; Conserved residues **UDPG (donor)** & **G6P (acceptor)** binding

TPP predicted domain; Conserved motifs **HAD phosphatases**

```
ZmTPSI.1.1_tps1/1-960 MSSDAARGQ---------RGINCTRGDAAAMPTSSPFVGDSGGAGSPIRVERMVRERSRR
ZmTPSI.1.2/1-576      ------------------------------------------------------------
ZmTPSII.2.1/1-888     ---MVSRSY---------SNLLELA------------AGGSGGEP-LPSLG--RRRIPRV
ZmTPSII.2.2/1-863     ---MASRSY---------SNLLDLA------------TGAADQAPAVAALGALRRRLPRV
ZmTPSII.3.1/1-912     ---MMSRSY---------TNLLDLAEGNFAALGP---VGGSGRQR-HGSFG--LRRMSRV
ZmTPSII.3.2/1-912     ---MMSRSY---------TNLLDLAEGNFAALGP---AGGSGRQR-HGSFG--LRRMSRV
ZmTPSII.3.3/1-912     ---MMSRSY---------TNLLDLAEGNFAALGPAAGAGGGGRQR-QGSFG--LRRMSRV
ZmTPSII.4.1/1-864     ---MVLKSH---------TNLLDMCCE----------DVFDFQQP--------LRSPRHV
ZmTPSII.4.2/1-865     ---MVSKSY---------SNLLDLTSG----------DGFDFRQP--------FKSLPRV
ZmTPSII.4.3/1-868     ---MVSKSY---------SNLLDMTPG----------DGFDFRRP--------FKSLPRV
ZmTPSII.5.1/1-953     MEAGASSRYPLWTRGGVQTRMEGMRGGVWKRRARM--EGRRRRRMAVGFAGGDLGRGARE
ZmTPSII.5.2/1-860     ------------------MTCLGHSCGPR--------ASGPCRAAIWPFVG--VQQINLK
ZmTPSII.5.3/1-986     ---MPSFPR---------ANVVDKTRGPSPCAHAVTPPTLTSGSPRPCPFLLPRRSDTHT
ZmTPSII.5.4/1-851     ---MPSISC---------HNLLDLA------------AADDVPLP-----SPTPLRLPRV

ZmTPSI.1.1_tps1/1-960 YDIFASDAMDTDAEAAFALDGVQSPGRA----------------------------SPAN
ZmTPSI.1.2/1-576      ------------------------------------------------------------
ZmTPSII.2.1/1-888     ---------------------VTASGIV----------------------------PDLD
ZmTPSII.2.2/1-863     ---------------------VTTPGLI----------------------------DD--
ZmTPSII.3.1/1-912     ---------------------MTVPGTL----------------------------TELD
ZmTPSII.3.2/1-912     ---------------------MTVPGTL----------------------------SELD
ZmTPSII.3.3/1-912     ---------------------MTVPGTL----------------------------SELD
ZmTPSII.4.1/1-864     ---------------------VNSPGII----------------------------SDPD
ZmTPSII.4.2/1-865     ---------------------VTSPGII----------------------------SDTD
ZmTPSII.4.3/1-868     ---------------------VTSPSII----------------------------SDHD
ZmTPSII.5.1/1-953     GGHRALCAYLENKKSGGPGWKIPLPACCAPALPSRGGPRPAVKYYDPIPRFQPHENPKSQ
ZmTPSII.5.2/1-860     ---------------------ATTGEVM--------------------------------
ZmTPSII.5.3/1-986     HIQLAIFVPPVLLPCPRPTSAFLSPETS-------------AKVIDPSPTPTPTDRPPNE
ZmTPSII.5.4/1-851     ---------------------MSVA-----------------------------------

ZmTPSI.1.1_tps1/1-960 MEDAGGAAA---------------------------------------------------
ZmTPSI.1.2/1-576      ------------------------------------------------------------
ZmTPSII.2.1/1-888     VSD---------------------------------------------------------
ZmTPSII.2.2/1-863     ------------------------------------------------------------
ZmTPSII.3.1/1-912     GEDESEPAA---------------------------------------------------
ZmTPSII.3.2/1-912     GEDESEPAA---------------------------------------------------
ZmTPSII.3.3/1-912     GEDESEPAA---------------------------------------------------
ZmTPSII.4.1/1-864     WES---------------------------------------------------------
ZmTPSII.4.2/1-865     WDT---------------------------------------------------------
ZmTPSII.4.3/1-868     WDS---------------------------------------------------------
ZmTPSII.5.1/1-953     TKRPNPCAA---------------------------------------------------
ZmTPSII.5.2/1-860     ------------------------------------------------------------
```

```
ZmTPSII.5.3/1-986     PDTPRRVAALLFSSCAIAAVSTSATPTDRQEEDEEAMPSLSCHNLLDLAAADEVPLPSPT
ZmTPSII.5.4/1-851     ------------------------------------------------------------

ZmTPSI.1.1_tps1/1-960 ----------ARPPLAGSRSGFRRLGLRGMKQRLLVVANRLPVSANRRGED-----HWSL
ZmTPSI.1.2/1-576      ------------------------------------------------------------
ZmTPSII.2.1/1-888     -----------AASAADQSS-------HAPRERVIIVANQLPVRASRRAAAGAGGGGWDF
ZmTPSII.2.2/1-863     --------------SPASPS-------TPPRPRTIIVANQLPIRSHRPESPEE---PWTF
ZmTPSII.3.1/1-912     ----------TSSVASDVPS-------SVAADRLIVVSNQLPIVARRRPDGR----GWSF
ZmTPSII.3.2/1-912     ----------TSSVASDVPS-------SVAVDRLIVVSNQLPIVARRRPDGR----GWSF
ZmTPSII.3.3/1-912     ----------TSSVASDAPS-------SVAADRLIVVSNQLPIVARRRPDGR----GWSF
ZmTPSII.4.1/1-864     -----------SNDGNSVGS------MPFCFKRKIIVANFLPVICAKNEATG----EWSF
ZmTPSII.4.2/1-865     -----------ISDGDSVGS-------ASSTERKIIVANFLPLNCTRDETG-----VLSF
ZmTPSII.4.3/1-868     -----------ISDGDSVGS-------AFSIERKIIVANFLPLNCTRDETG-----ELSF
ZmTPSII.5.1/1-953     ----------QAAAMLSVSASDGDRSPCPVEARRIVVTHRLPLHAEPNPDAPY---GFDF
ZmTPSII.5.2/1-860     -----------VSKTSSPSASNGELSTSPVEARRIVVTYRLPLRAEPNLDSPH---GFDF
ZmTPSII.5.3/1-986     PLRLPRVMSVASPASPTSPS-------PPAPPRRVIVSHRLPLRASPDPSAPF---GFRF
ZmTPSII.5.4/1-851     -----------SPASPTSPP-------APAPPRRVIVSHRLPLRASPDPAAPF---GFAF


ZmTPSI.1.1_tps1/1-960 EISAGGLVSALL-------GVKDVDAKWIGWAGVNVPDEVGQRALTKALAEK-RCIPVFL
ZmTPSI.1.2/1-576      ------------------------------------------------------------
ZmTPSII.2.1/1-888     AWDQDSLLLQVKDSLRAHHGRADVEFVYVGGLRDDVPP-AEHDQVAHDLLEGFRCVPTFL
ZmTPSII.2.2/1-863     EWDEDSLLRHLH-----HSSSPLMEFIYIGCLRDDIPQ-AEQDAVAQALLETHNCVPAFL
ZmTPSII.3.1/1-912     SWDDDSLLLQLR-----DGIPDEMEVLFVGSLRADVPA-AEQDAVSQALLDRFRCAPVFL
ZmTPSII.3.2/1-912     SWDGDSLLLQLR-----DGIPDEMEVLFVGSLRADVPA-AEQDEVSQTLLDRFRCAPVFL
ZmTPSII.3.3/1-912     VWDDDSLLLQLR-----DGIPEDMEVLFVGSLRADVPV-AEQDEVSQALLDRFRCAPVFL
ZmTPSII.4.1/1-864     AMDDNQLLVQLK-----DGFPIGNEVIYVGSLNVQVDP-IEQDRVSQKLFKEHRCVPTFL
ZmTPSII.4.2/1-865     SLDHDALLMQLK-----DSFSNETDVVYVGSLKVQVDP-GEQDQVAQKLLREYRCIPTFL
ZmTPSII.4.3/1-868     SLDHDSLLMQLK-----DGFSNETDAVYVGSLKVHVDP-REQDQVAQKLLREYRCIPTFL
ZmTPSII.5.1/1-953     SLDADALPLQLA-----RGLPR--PVVFVGALPSAAASISASEELEADLLARFGCSPVFL
ZmTPSII.5.2/1-860     SLDADALPLQCT-----RGLPR--PVVFVGALPSAAASISESDDLAADLFTRFACSPVFL
ZmTPSII.5.3/1-986     SVDAGTVAYQLR-----SGLPTNAPVLHIGTLPASAAE-AASDELSNYLLANFSCLPVYL
ZmTPSII.5.4/1-851     SVDAGTVAYQLR-----SGLPANAPVLHIGTLPAAAAE-AASDELSDYLLANFSCLPVYL

ZmTPSI.1.1_tps1/1-960 DEEIVHQYYNGYCNNILWPL--FHYLGLPQEDRL----------------ATTRNFESQF
ZmTPSI.1.2/1-576      ------------------------------------------------------------
ZmTPSII.2.1/1-888     PADLRSRFYHGFCKQQLWPL--FHYM-LPLSPEL-----------------GGRFDRLLW
ZmTPSII.2.2/1-863     PTDIAERYYHGFCKQHLWPL--FHYM-LPLSPDL-----------------GGRFDRALW
ZmTPSII.3.1/1-912     PDHLNDRFYHGFCKRQLWPL--FHYM-LPFSSPASASAAATSSSVATSSPGNGCFDRSAW
ZmTPSII.3.2/1-912     PDHLNDRFYHGFCKRQLWPL--FHYM-LPFSSPASASAAATSSSVATSSPGNGRFDRSAW
ZmTPSII.3.3/1-912     PDRLNDRFYHGFCKRQLWPL--FHYM-LPFSSSA---SAAGTTSSSSAATCNGRFDRSAW
ZmTPSII.4.1/1-864     PAELQHQFYHIFCKQHLWPL--FHYM-LPVCHDK-----------------DELFDRSLF
ZmTPSII.4.2/1-865     PSDLQQQFYHGFCKQQLWPL--FHYM-LPICLDK-----------------GELFDRSLF
ZmTPSII.4.3/1-868     PSDLQQQFYHGFCKQQLWPL--FHYM-LPICLDK-----------------GELFDRTLF
ZmTPSII.5.1/1-953     DPGLHKDFYDGFCKRYLWPM--LHYL-LPFTLTP------------FFGSGGLKFKANLY
ZmTPSII.5.2/1-860     DPSLHNDFYNSFCKRYLWPILQLQYL-LPFTRSS--------------DSGCLSFNEDLY
ZmTPSII.5.3/1-986     PTDLHHRFYHGFCKHYLWPL--LHYL-LPLTPSS---------------LGGLPFQRTLY
```

```
ZmTPSII.5.4/1-851     PTDLHHRFYHGFCKHYLWPL--LHYL-LPLTPSS---------------LGGLPFQRTLY

ZmTPSI.1.1_tps1/1-960 DAYKRANQMFADVVYEHYQ-DGDVIWCHDYHLMFLPKCLKDHDINMKVGWFLHTPFPSSE
ZmTPSI.1.2/1-576      ------------------------------------------------------------
ZmTPSII.2.1/1-888     QAYVSVNKIFADKILEVISPDEDFVWVHDYHLMVLPTFLRKRFNRVKLGFFLHSPFPSSE
ZmTPSII.2.2/1-863     QAYVSANKIFADKVLEVINPDDDFVWVHDYHLMVLPTFLRKRFNRIKLGFFLHSPFPSSE
ZmTPSII.3.1/1-912     EAYVLANKFFFEKVVEVINPEDDYVWVHDYHLLALPTFLRRRFNRLRIGFFLHSPFPSSE
ZmTPSII.3.2/1-912     EAYVLANKFFFEKVVEVINPEDDYVWVHDYHLLALPTFLRRRFNRLRIGFFLHSPFPSSE
ZmTPSII.3.3/1-912     EAYVLANKFFFEKVVEVINPEDDYVWVHDYHLMALPTFLRRCFNRLRIGFFLHSPFPSSE
ZmTPSII.4.1/1-864     QAYVRANKIFADKIVEAVNSDDDCVWVHDYHLMLIPTLLRKKLHRIKVGFFLHSPFPSSE
ZmTPSII.4.2/1-865     QAYVRANKLFADKVMEAINADDDFVWVHDYHLMLLPTFLRKRLHRIKIGFFLHSPFPSSE
ZmTPSII.4.3/1-868     QAYVRANKLFADKVMEAINTDDDYVWVHDYHLMLLPTFLRKRLHRIKIGFFLHSPFPSSE
ZmTPSII.5.1/1-953     RAYLTANTQYAERVLEQLNPDEDLVFIHDYHLLALPTILRHKSPRARIGFFLHTPFPTSE
ZmTPSII.5.2/1-860     RAYLTANTQYADRVFEHLNTDEDLVLIHDYHLFALPTILRRKSPRARIGFFLHSPFPTSE
ZmTPSII.5.3/1-986     HSFLSANRAFADRLTEVLSPDEDLVWIHDYHLLALPTFLRKRFPRAKVGFFLHSPFPSSE
ZmTPSII.5.4/1-851     HSFLSANRAFADRLTEVLCPDEDLVWIHDYHLLALPTFLRKRFPRAKVGFFLHSPFPSSE

ZmTPSI.1.1_tps1/1-960 IYRTLPSRLELLRSVLCADLVGFHTYDYARHFVSACTRILGLEGTPE----GVEDQGRLT
ZmTPSI.1.2/1-576      ------------------------------------------------------------
ZmTPSII.2.1/1-888     IYKTLPVREELLRSLLNADLIGFHTFDYARHFLSCCSRMLGLKYESQRGYIALEYYGRTV
ZmTPSII.2.2/1-863     IYKTLPVREELLRALLNSDLIGFHTFDYARHFLSCCGRMLGLSYESKRGHICLEYYGRTV
ZmTPSII.3.1/1-912     IYRTLPVRDEILKALLNCDLIGFHTFDYARHFLSCCSRMLGIEYQSKRGYIGLDYFGRTV
ZmTPSII.3.2/1-912     IYRTLPVRDEILKALLNCDLIGFHTFDYARHFLSCCSRMLGIEYQSKRGHIGLDYFGRTV
ZmTPSII.3.3/1-912     IYRTLPVREEILKALLNCDLIGFHTFDYARHFLSCCSRMLGIEYQSKRGYIELDYFGRTV
ZmTPSII.4.1/1-864     IYRTLPVRDEILKSLLNADLIGFQTFDYARHFLSCCSRLLGLNYESKRGHIGIEYFGRTV
ZmTPSII.4.2/1-865     IYRTLPVRDEILKSLLNADLIGFQTFDYARHFLSCCSRLLGLHYESKRGYIGIEYFGRTV
ZmTPSII.4.3/1-868     IYRTLPVRDEILKSLLNADLIGFQTFDYARHFLSCCSRLLGLHYESKRGYIGIEYFGRTV
ZmTPSII.5.1/1-953     LFRTVPVREDLLRSLLNADLVGFHNYDYARHFLSACTRLLGVTSHTHRGYISIDYCGRAV
ZmTPSII.5.2/1-860     LFRAIPVREELLRALLNADLVGFQNYDYGCHFISACSTLLGITSRAHGDYICIDYFGRAV
ZmTPSII.5.3/1-986     IFRTIPVRDDLVRALLNADLVGFHTFDYARHFLSACSRLLGLDYQSKRGYIGIEYYGRTV
ZmTPSII.5.4/1-851     IFRTIPVRDDLVRALLNADLVGFHTFDYARHFLTACSRLLGLDYQSKRGYIGIEYYGRTV

ZmTPSI.1.1_tps1/1-960 RVAAFPIGIDSDRFKRALELPAVKRHVSELTERF--AGRKVMLGVDRLDMIKGIPQKILA
ZmTPSI.1.2/1-576      -----------------------------------------MLGVDRLDMIKGIPQKILA
ZmTPSII.2.1/1-888     TIKILPVGVHLEQLRSVLNLPELGVKVAELLKQFCHRNRLLLLGVDDMDIFKGISLKLLA
ZmTPSII.2.2/1-863     SIKILPVGVHMEQLKTVLGLPETEAKVSELMEMYSGKGRVVMLGVDDMDIFKGISLKLLA
ZmTPSII.3.1/1-912     GIKIMPVGVHMGQLESGLRLPDREWRLSELQQQF--QGKTVLLGVDDMDIFKGINLKLLA
ZmTPSII.3.2/1-912     GIKIMPVGVHMGQLESGLRLPDREWRLSELQQQF--QGKTVLLGVDDMDIFKGINLKLLA
ZmTPSII.3.3/1-912     GIKIMPVGVHMGQLELGLRLPDREWRLSELQRQF--QGKTVLLGVDDMDIFKGINLKLLA
ZmTPSII.4.1/1-864     SLKILAAGVHVGRLEATLRLPATIKKVQEIESRY--SGKLVILGVDDMDIFKGISLKLLG
ZmTPSII.4.2/1-865     SLKILSVGVHIGRLESVLKLPATVSKVQEIEQRY--KGKILMLGVDDMDIFKGISLKFLG
ZmTPSII.4.3/1-868     SLKILSVGVHVGRLESVLKLPATVSKVEEIEQRY--KGKILMLGVDDMDIFKGISLKLLA
ZmTPSII.5.1/1-953     SVKILAGGVDIGQLREVLSSPETEAKAKEVATKF--AGRQLLLGVDDVDLFKGIGLKLLA
ZmTPSII.5.2/1-860     VVKILSVGVDMVRLREVLSSPETAAKAKEVATKF--AGRQVLIGVDDVDLFNRIDVKLLA
ZmTPSII.5.3/1-986     TVKILPVGIDMGQLRSVVSAPETEDAVRRVTEAY--KGRRLMVGVDDVDLFKGIGLKFLA
ZmTPSII.5.4/1-851     TVKILPVGIDMGQLRSVVSAPETEDAVRRVTEAY--KGRRLMVGVDDVDLFKGIGLKFLA
```

```
ZmTPSI.1.1_tps1/1-960 FEKFLEENPDWNNKVVLLQIAVPTRTDVPEYQKLTSQVHEIVGRINGRFGTL-TAVPIHH
ZmTPSI.1.2/1-576      FEKFLEENPDWNDKVVLLQIAVPTRTDVPEYQKLTSQVHEIVGRINGRFGTL-TAVPIHH
ZmTPSII.2.1/1-888     FEQLLMQHPEWRGRVVLVQIANPARGRGKDVREVQEESDAMVRRINDAFGQP-GYQPVIL
ZmTPSII.2.2/1-863     MEELLRQHPEWRGKLVLVQVANPARGRGKDVAEVQTETYAMVRRINEVYGEP-GYEPVVL
ZmTPSII.3.1/1-912     FENMLRTHPKWQGRAVLVQIANPARGRGKDLEAIQAEIEQSCQRINVDFGQS-GYSPVVF
ZmTPSII.3.2/1-912     FENMLRTHPKWQGRAVLVQIANPARGRGKDLEAIQAEIEESCQRINGDFGQS-GYSPVVF
ZmTPSII.3.3/1-912     FENMLRTHPKWQGRAVLVQIANPARGRGKDLEAIQAEIEESCQRINGDFGQS-GYSPVVF
ZmTPSII.4.1/1-864     LELLLERTPKLRGKVVLVQIVNPARSIGKDIEEAKYEAESVAQRINDKYGSA-NYKPVVL
ZmTPSII.4.2/1-865     LELLLDRNPKLREKVVLVQIINPARSTGKDVQEAITEAVSVAERINTNYGSS-SYKPVVL
ZmTPSII.4.3/1-868     LELLLDRNPKLREKVVLVQIINPARSTGKDVQEAITEAVSVAERVNTKYGSS-SYKPVVL
ZmTPSII.5.1/1-953     MERLLESQPELHGQVVLVQINNPVRSPGYDTDEICAELQAMRKRINARFATPAGYEPIVI
ZmTPSII.5.2/1-860     MERLLESRPELIGQVVLVQINNPARSPGRDTDTVLAEVQLLMDRINARFAKP-GYDPIVM
ZmTPSII.5.3/1-986     MEQLLVEHRELRGRAVLVQIANPARSEGRDVQGVQDEARAISARVNARFGTP-GYTPIVL
ZmTPSII.5.4/1-851     MEQLLVEHRELRGHAVLVQIANPARSEGRDVQGVQDEARAISARVNARFGTP-GYTPIVL

ZmTPSI.1.1_tps1/1-960 LDRSLDFHALCALYAVTDVALVTSLRDGMNLVSYEYVACQGS--------------KKG
ZmTPSI.1.2/1-576      LDRSLDFHALCALYAVTDVALVTSLRDGMNLVSYEYVACQGS--------------KKG
ZmTPSII.2.1/1-888     IDQPLQFYERMAYYVVAECCLVTAVRDGMNLIPYEYVIARQGNERIDSILGLGPASRKKS
ZmTPSII.2.2/1-863     IDEPLQFYERVAYYVIAEVCLVTAVRDGMNLIPYEYIVSRQGNEKLDRMLRQGKPEEKKS
ZmTPSII.3.1/1-912     INRDVSSVEKVAYYTIAECVVVTAVRDGMNLTPYEYIVCRQGAPGSESVSEVSGP--KKS
ZmTPSII.3.2/1-912     IDRDVSSVEKIAYYTIAECVVVTAVRDGMNLTPYEYIVCRQGAPGSESVSEVGGP--KKS
ZmTPSII.3.3/1-912     IGRDVSSVEKIAYYTIAECVVVTAVRDGMNLTPYEYVVCRQGAPGSQSVSEVSGP--KKS
ZmTPSII.4.1/1-864     IDYSIPFYEKIAFYAASDCCIVNAVRDGMNLIPYEYTVCRQGNEELDKLRGLNKSSSHTS
ZmTPSII.4.2/1-865     IDHHIPFYEKIAFYAASDCCIVNAVRDGMNLVPYEYTVCRQGNEEIDKLRGLGKDTHHTS
ZmTPSII.4.3/1-868     IDNRIPFYEKVAFYAASDCCIVNAVRDGMNLVPYEYTVCRQGNEEIDRVRGLDKDTHHTS
ZmTPSII.5.1/1-953     IEDPMTMHEKLAFYTSADICLVTAVRDGLNRTPYIYTVCRQEGPISSGVVGAP----KEG
ZmTPSII.5.2/1-860     IDDPLTMHEKLAFYTSADICIVTAVRDGLNRTPYIYTVCREHGPISSGVAGAP----RES
ZmTPSII.5.3/1-986     IDGPVTPQEKAAYYAAAECCVLSAVRDGLNRIPYIYTVCRQES------TALGDDAPKRS
ZmTPSII.5.4/1-851     IDAPVTPQEKAAYYAAAECCVVSAVRDGLNRIPYIYTVCRQES------TALGDDSPKRS

ZmTPSI.1.1_tps1/1-960 VLILSEFAGAAQSLGAGAILVNPWNITEVADSIRHALTMPSDEREKRHRHNYAHVTTHTA
ZmTPSI.1.2/1-576      VLILSEFAGAAQSLGAGAILVNPWNITEVADSIHHALTMPSDEREKRHRHNYAHVTTHTA
ZmTPSII.2.1/1-888     MLVVSEFIGCSPSLS-GAIRVNPWNIDSVADAMDYALEMPEGEKVLRHEKHHRYVSTHDV
ZmTPSII.2.2/1-863     MLVVSEFIGCSPSLS-GAIRVNPWNIEAVADAMETALVLPENEKRLRHDKHFRYVSTHDV
ZmTPSII.3.1/1-912     MLVVSEFIGCSPSLS-GAIRVNPWNIEATAEAMNEAISMPEQEKQLRHEKHYRYVSSHDV
ZmTPSII.3.2/1-912     MLVVSEFIGCSPSLS-GAIRVNPWNIEATAEAMNEAISMPEQEKQLRHEKHYRYVSSHDV
ZmTPSII.3.3/1-912     MLVVSEFIGCSPSLS-GAIRVNPWNIEATAEAMNEAISMPEQEKQLRHEKHYRYVRSHDV
ZmTPSII.4.1/1-864     TLIVSEFVGCSPSLS-GAFRVNPWSMEDVADALYSVTDLTRYEKNLRHEKHYRYVRSHDV
ZmTPSII.4.2/1-865     TLIVSEFVGCSPSLS-GAFRVNPWSVDDVADALCRATDLTESEKRLRHEKHYRYVSTHDV
ZmTPSII.4.3/1-868     TLIVSEFVGCSPSLS-GAFRVNPWSVDDVADALCRATDLSESEKRLRHEKHYRYVSTHDV
ZmTPSII.5.1/1-953     AIVLSEFVGCATSLG-GAVHINPWNVDAVAEGMHMALRFNGREKQVRQEKHYRFVSTHDI
ZmTPSII.5.2/1-860     AIVLSELVGCSTFLR-GAVRVNPWNVDDVVEGMSSALRLNERDKKILHAKHYMYVVKHDI
ZmTPSII.5.3/1-986     AIVLSEFVGCSPSLS-GAIRVNPWSVESVAEAMNAALRMPEAEQRLRHEKHYKYVSTHDV
ZmTPSII.5.4/1-851     VIVLSEFVGCSPSLS-GAIRVNPWSVESVAEAMNAALRMPEAEQRLRHEKHYKYVSTHDV
```

```
ZmTPSI.1.1_tps1/1-960 QDWAETFVFELNDTVAEAL------------LRTRQVPPG---LPSQMAIQQYLRSKNRL
ZmTPSI.1.2/1-576      QDWAETFVFELNDTVAEAL------------LRTRQVPPG---LPGQTAIQQYLRSKNRL
ZmTPSII.2.1/1-888     GYWANSFLQDLERICLDHNRRRCWGIGFGLKFRVVALDPNFKKLAVEHLVLAYRRTKKRV
ZmTPSII.2.2/1-863     GYWANSFLLDLERTCKYHSQKRCWGIGFGLRFRVVSLDLTFRKLSLENILMAYRRAKTRA
ZmTPSII.3.1/1-912     AYWSKSFILDLERACRDHFKRTCWGIGLGFGFRVVALDPHFRKLNMDSIVNAYEISESRA
ZmTPSII.3.2/1-912     AYWSKSFILDLERACRDHFKRTCWGIGLGFGFRVVALDAHFRKLNMDSIVNAYEISGSRA
ZmTPSII.3.3/1-912     AYWSKSFIIDLERVCKDHFKRTCWGIGLGFGFRVVALDPHFTKLNMDSIINAYELSESRA
ZmTPSII.4.1/1-864     AYWARSFDQDLDKACIEQYSQRCWTTGFGLNFRVIALSPGFRRLSLEHLASSYKKANRRM
ZmTPSII.4.2/1-865     AYWARSFAQDLERACKDHYSRRCWAIGFGLNFRVIALSPGFRKLSSEHFVSSYNKASRRA
ZmTPSII.4.3/1-868     AYWAHSFAQDLERACRDHYSRRCWAIGFGLNFRVIALSPGFRKLSSEHFVSSYNRASRRA
ZmTPSII.5.1/1-953     AYWGRSLDQDLQRASKDHASMKFMSVGLAMSYHIVVLSPNFQKLSPEHINPSYQRAGNRL
ZmTPSII.5.2/1-860     AYWGRSLDQNLQKASMDHASMNFLSVGLAMNFRIVVLDPNFQKLSPEHINPSYHRTGNRL
ZmTPSII.5.3/1-986     AYWARSFDSDLQRACKDHFSRRHWGIGFGMSFKVVALGPNFRRLSVEHIVPSYRRTENRL
ZmTPSII.5.4/1-851     AYWARSFDSDLQRACKDHFSRRHWGIGFGMSFKVVALGPNFRRLSVEHIVPSYRRTDNRL

                        MOTIF I                                            MOTIF II
ZmTPSI.1.1_tps1/1-960 LILGFNSTLTEPVESSGRRGGDQIKEMELKLHPDLKGPLRALCEDERTTVIVLSGSDRSV
ZmTPSI.1.2/1-576      LILGFNSTLTEPVESSGRRGGDQIKEMELKLHPDLKGPLGALCEDERTTVIVLSGSDRSV
ZmTPSII.2.1/1-888     ILLDYDGTLM-PQTSLGKSPTSRTIDM-----------LNSLCRDRNNMVFLVSAKSRMT
ZmTPSII.2.2/1-863     ILLDYDGTLM-PQ-AINKSPSTESVRI-----------LNSLCRDKDNVVYLCSGYDRRT
ZmTPSII.3.1/1-912     ILLDYDGTLV-PQTSINKEPSPEVLNI-----------INTLCSDSRNIVFLVSGRDKDT
ZmTPSII.3.2/1-912     ILLDYDGTLV-PQTSINKEPSPEVLNI-----------INTLCSDSRNIVFLVSGRDKDM
ZmTPSII.3.3/1-912     ILLDYDGTLV-PQTSLNKEPSPQVLSI-----------INTLCSDSRNIVFLVSGRDKDT
ZmTPSII.4.1/1-864     IFLDYDGTLV-PQTSHDKSPSAELIST-----------LNSLCSDMKNTVFIVSGRGRDS
ZmTPSII.4.2/1-865     IFLDYDGTLV-PQSSINKAPSEEVISV-----------LNTLCNDPKNIVFIVSGRGRDS
ZmTPSII.4.3/1-868     IFLDYDGTLV-PQSSINKAPSEEVISI-----------LNTLCNDPKNVVFIVSGRGRDS
ZmTPSII.5.1/1-953     ILLDYDETVMFHPGLLDRHPSQRLIGI-----------LNELCSDPKNTVFVVSGRSKDE
ZmTPSII.5.2/1-860     ILLDYDGTVMVPQGLITRHPSQELVSV-----------LNELCSDPMNTVFVVSGRSKDE
ZmTPSII.5.3/1-986     ILLDYDGTVM-PENSIDRTPSSEVISV-----------LNRLCEDPKNRVFIVSGRGKDE
ZmTPSII.5.4/1-851     ILLDYDGTVM-PENSIDRTPSSEVISV-----------LNRLCEDPKNRVFIVSGRGKDE

ZmTPSI.1.1_tps1/1-960 LDENFGEF-KMWLAAEHGMFLR-PTYGEWMTTMPEHLNMDWVDSVKHVFEYFTERTPRSH
ZmTPSI.1.2/1-576      LDENFGEF-KMWLAAEHGMFLR-PTYGEWMTTMPEHLNMDWVDSVKHVFEYFTERTPRSH
ZmTPSII.2.1/1-888     LNEWFLPCESLGLAAEHGCFLRLRRDAEWETCVPV-IDCSWKQIAEPVMKTYTETTDGST
ZmTPSII.2.2/1-863     LHEWF-PCENLGIAAEHGYFLRCKRDAEWKTCVAA-TDCSWKQIAEPVMCLYRETTDGST
ZmTPSII.3.1/1-912     LGKWFSSCPKLGIAAEHGYILRWSSKEEWQTCTQA-MDFGWMQMAKPVMNLYTEATDGSY
ZmTPSII.3.2/1-912     LGKWFSSCPKLGIAAEHGYFLRWSSEEEWQTCTQA-MDFGWMQMAKPVMNLYTEATDGSY
ZmTPSII.3.3/1-912     LGKWFSSCPRLGIAAEHGYFLRWSREEEWQTCTQA-LDFGWMQMAKPVMNLYTEATDGSY
ZmTPSII.4.1/1-864     LSEWFASCENLGIAAEHGYFIRWNKAAEWETSFSG-IYSEWKLIADPIMHVYMETTDGSF
ZmTPSII.4.2/1-865     LDEWFSPCEKLGLAAEHGYFIRWSKEAAWESSYSR-PQQEWKHIAEPVMQVYTETTDGSS
ZmTPSII.4.3/1-868     LDEWFSPCEKLRLAAEHGYFIRWSKEAAWESSYSS-PRQEWKHIAEPVMQVYTETTDGSS
ZmTPSII.5.1/1-953     LARWLEPCERLGISAEHGYFTRWSRYSPWESPDLK-VDYGWKKMVEPVMDLYVAVTDGSS
ZmTPSII.5.2/1-860     LAGWLAPCEKLGISAEHGYFTRWSRDSPWESPKLL-LDNDWKNIVEPVMKYYCDVTDGSY
ZmTPSII.5.3/1-986     LSRWFAPCEKLGIAAEHGYFTRWSRDAPWEASALA-ADLDWKNTAEPVMRLYTEATDGSY
ZmTPSII.5.4/1-851     LSRWFAPCEKLGIAAEHGYLTRWSRDAPWDTSGLA-ADFDWKKTAEPVMQLYTEATDGSY

ZmTPSI.1.1_tps1/1-960 FEHRETSFVWNYKYADVEFGRLQARDMLQHLWTGPISNAAVDVVQGSRSVEV--RSVG-V
ZmTPSI.1.2/1-576      FEHRETSFVWNYKYADVEFGRLQARDMLQHLWTGPISNAAVDVVQGSRSVEV--RSVG-V
ZmTPSII.2.1/1-888     IEDKETAIVWCYEDADPDFGSCQAKELHDHL-ESVLANEPVSVKAGPNLVEV--KPQG-V
```

```
ZmTPSII.2.2/1-863     IEDRETVLVWNYEDADPDFGSCQAKELVDHL-ESVLANEPVSVKTTPHSVEV--KPQG-V
ZmTPSII.3.1/1-912     IERKESALVWHHQDADPGFGSSQAKELLDHL-ESVLANEPVSVKSGQFIVEV--KPQG-V
ZmTPSII.3.2/1-912     IETKESALVWHHQDADPGFGSSQAKELLDHL-ESVLANEPVSVKSGQFIVEV--KPQG-V
ZmTPSII.3.3/1-912     IEAKESALVWHHQDADLGFGSSQAKEMLDHL-ESVLANEPVSVKSGQFIVEV--KPQG-I
ZmTPSII.4.1/1-864     IEPKESALVWHYQNTDHDFGSCQAKELVSHL-ERVLSNEPVVVRRGHQIVEV--KPQG-V
ZmTPSII.4.2/1-865     IESKESALVWHYLDADHDFGSFQAKELQGHL-ERVLSNEPVVVKCGHYIVEV--KPQG-V
ZmTPSII.4.3/1-868     VESKESALVWHYLDADHDFGSFQAKELKDHL-ERVLSNEPVVVKCGHYIVEV--KPQG-V
ZmTPSII.5.1/1-953     VETKETALVWHYEGTDPVFGPSQAKELRDHL-SDVLAKEPVSVRSGYNIVEV--NPQE-V
ZmTPSII.5.2/1-860     IEAKETALVWHYEEADPVFGPRQAKELQYHL-RDVLSEEPVYVKSGHQIVEVNGNPQEVV
ZmTPSII.5.3/1-986     IEHKESGMVWHHDEADPDFGSCQAKELLDHL-ENVLANEPVVVKRGQHIVEV--NPQG-I
ZmTPSII.5.4/1-851     IEHKESAIVWHHHEADPDFGSCQAKELLDHL-ENVLANEPVVVKRGQHIVEV--NPQG-I


                                  MOTIF III
ZmTPSI.1.1_tps1/1-960 TKGAAIDRILGEIV-HSENMITP-IDYVLCIGHFLGKDEDIYVFFDPEYPSESKVKPEGG
ZmTPSI.1.2/1-576      TKGAAIDRILGEIV-HSENMITP-IDYVLCIGHFLGKDEDIYVFFDPEYPSESKVKPEGG
ZmTPSII.2.1/1-888     SKGLVAKRILSTTQ-ERGDADDDLPDFVLCVGD-DRSDEDMF---------EVIAAAAA-
ZmTPSII.2.2/1-863     SKGLVARRMLVSMK-ERGQC----PDFVLCIGD-DKSDEDMF---------QLIATAAC-
ZmTPSII.3.1/1-912     SKGIVAERILASVK-ERGKQ----ADFVLCIGD-DRSDEDMF---------ENIADIIK-
ZmTPSII.3.2/1-912     SKGIVAERILASVK-ERGKQ----ADFVLCIGD-DRSDEDMF---------ENIADIIK-
ZmTPSII.3.3/1-912     SKGIVAERILASVK-ERGKQ----ADFLLCIGD-DRSDEDMF---------ENIADIIG-
ZmTPSII.4.1/1-864     SKGISVDKIIRTLV-SKGEV----PDLLMCIGN-DRSDEDMF---------ESINRATS-
ZmTPSII.4.2/1-865     SKGLAVNKLIHTLV-KNGKA----PDFLMCVGN-DRSDEDMF---------ESINGMTS-
ZmTPSII.4.3/1-868     SKGRAVDKLIQALANNNGKA----QDFLMCVGN-DRSDEDMF---------ECINGMAS-
ZmTPSII.5.1/1-953     DKGTAVQRIIAAMR-DRGRM----PDFILCVGD-DASDEDMF---------KAVTAPSN-
ZmTPSII.5.2/1-860     GKGTAVQGLIAALG-ARGRM----PDFILCVGD-DVSDEDMF---------EAISAPSS-
ZmTPSII.5.3/1-986     SKGVVVDSLLSSMV-RTGKP----PDFVLCIGD-DRSDEDMF---------ESIVCPAS-
ZmTPSII.5.4/1-851     SKGVVVDSLLSSMV-RTGKP----PDFVLCIGD-DRSDEDMF---------ESIVCPAS-
                                                                     MOTIF III
ZmTPSI.1.1_tps1/1-960 SASLDRRPNGRPPSNGRSNSRNPQSRTQKAQQAASERSSSSSHSSTSSNHDWREGSSVLD
ZmTPSI.1.2/1-576      SASLDRRPNGRPASNGRSNSRNPQSRPQKAQQAASERSSSSSHSSTSSNHDWREGSSVLD
ZmTPSII.2.1/1-888     ----------------------------------------------------ARGVSSLQ
ZmTPSII.2.2/1-863     ----------------------------------------------------G---DSLA
ZmTPSII.3.1/1-912     ----------------------------------------------------R---NMVA
ZmTPSII.3.2/1-912     ----------------------------------------------------R---NMVA
ZmTPSII.3.3/1-912     ----------------------------------------------------R---NLVA
ZmTPSII.4.1/1-864     -------------------------------------------------------LSELP
ZmTPSII.4.2/1-865     ----------------------------------------------------N---AVLS
ZmTPSII.4.3/1-868     ----------------------------------------------------N---DVSS
ZmTPSII.5.1/1-953     ----------------------------------------------------K---SAFP
ZmTPSII.5.2/1-860     ----------------------------------------------------KF---AFP
ZmTPSII.5.3/1-986     ----------------------------------------------------SSGGVRLP
ZmTPSII.5.4/1-851     ----------------------------------------------------NSG-VKLP


ZmTPSI.1.1_tps1/1-960 LKGENYFSCAVGRKRSNARYLLSSSEEVVSFLKELATA--------------------TA
ZmTPSI.1.2/1-576      LKAENYFSCAVGRKRSNARYLLSSSEEVVSFLKELATE--------------------TA
ZmTPSII.2.1/1-888     AEA-EVFACTVGRKPSKAKYYLDDPADIVRLVQGLASV--------SDDDQTHAPPPPPP
ZmTPSII.2.2/1-863     SKA-EVFACTVGRKPSKAKYYLDDAAEVVRLMQGLSYV-----------SEELALANQRD
```

```
ZmTPSII.3.1/1-912      PRT-SLFACTVGQKPSKAKFYLDDTFEVVAMLSALADATGAELKSDSADELAASISSLDI
ZmTPSII.3.2/1-912      PRT-SLFACTVGQKPSKAKFYLDDTFEVVAMLSALADATGAELESDSADELAASISSLDI
ZmTPSII.3.3/1-912      PRT-ALFACTVGQKPSKAKFYLDDTFEVVTMLSALADATGPELETDSADESVAYISSLDI
ZmTPSII.4.1/1-864      AAP-EVFACSVGPKASKANYYVDGCDEVIRLLKGVTAV---------------SLQKDTA
ZmTPSII.4.2/1-865      PTMPELFACSVGQKPSKAKYYVDDTSEVIRLLKNVTRI-------------PSQRQDVSA
ZmTPSII.4.3/1-868      TTVPEVFACSVGQKPSKAKYYVDDTSEVIRLLRDATRF-----------SSSQRREDVNA
ZmTPSII.5.1/1-953      EDA-EVFACTIGTKPSLAKYYLDDPVEVLSMLKGLIKS----------------SVEERP
ZmTPSII.5.2/1-860      EAA-EIFACTVGNKPSLAKYYLEDPDEVLKMLKGLIDS-------------------FEE
ZmTPSII.5.3/1-986      ASS-EVFACTVGKKPSMAKYYLDDTVDVVKMLDGLASA--------------PSPPRRPG
ZmTPSII.5.4/1-851      ASS-EVFACTVGKKPSMARYYLDDTVDVVKMLDGLASA-----------------PSQQR

ZmTPSI.1.1_tps1/1-960 GFQATCADYMHVLG-
ZmTPSI.1.2/1-576       GFQSSCADYMFLDRQ
ZmTPSII.2.1/1-888      SAATTAADTVPR---
ZmTPSII.2.2/1-863      EDEDSSLDDVWE---
ZmTPSII.3.1/1-912      GDEQSESSDTPIGGS
ZmTPSII.3.2/1-912      GDEQSETSDTPIGGS
ZmTPSII.3.3/1-912      GDEQSESSDKPVEGS
ZmTPSII.4.1/1-864      GHSHAAFEDTLEVVS
ZmTPSII.4.2/1-865      SHGRVTFRGVLDYVD
ZmTPSII.4.3/1-868      SRGRVTFRDALDYVD
ZmTPSII.5.1/1-953      GDGEGPSRVSFD---
ZmTPSII.5.2/1-860      NHSTVEANK------
ZmTPSII.5.3/1-986      PAAAVQLRVSFEGSL
ZmTPSII.5.4/1-851      SRPAVQLRVSFEGSL
```

Supplemental Figure S2B. Predicted enzymatic domains for TPP proteins.

TPP predicted domain; Conserved motifs **HAD phosphatases**

ZmTPPA.1/1-388 --MDLKTGLNS---PVIADHLPTLALPAA-VMTFTTPTSFP---SP--------------

ZmTPPA.3/1-369 --MDMGSG-SS---PVITDPISISPPLLGGLTSNLMPFSVM---SG--------------

ZmTPPB.1.1/1-367 --MTKQGMVVPV--PEAAVAVPPNSAP---LFQYPPPRAAP---GV--------------

ZmTPPB.1.2/1-384 MPMAKPSVAVAEASGVPAQASCSCPCPGTTLFPYPPPRGASGIAAA--------------

ZmTPPB.1.3/1-370 --MAKPSVAVPEV-GVPAAQA-SCTCPGT-LLAYPPPRGAG--VAA--------------

ZmTPPB.1.4/1-357 -MTNQQDVVVSEM-GIAAGAALPGPSPA--LLAC---RGAA---AG--------------

ZmTPPB.1.5/1-356 --MTNQDVVVSEM-GIAAGTALPGSSPA--LLAC---RGAA---AG--------------

ZmTPPB.1.6/1-384 --MTNQDVVVPDM-GIAAAAALP--PPG--LFACRGVAGAVSSLRGTYGSLGLPGGAAAD

ZmTPPB.2.1_ramosa3/1-361 --MTKHAAYSSED-VVAAVAAPA--PAGRHFTSFQALKGAP----L--------------

ZmTPPB.2.2/1-327 --MTKHTAFAGADGGTTAAAA---------VTLCAPPRA-----RG--------------

ZmTPPB.2.3/1-357 --MTKRTAFAADD-AIIAAAAAVTSQPGRRFTSYPPARA-----RG--------------

ZmTPPA.1/1-388 GLCLNTTKKIPLPGKIEEVRAAG-W-LDLMKASSPTRKRQIKDVICDAQSD---LDLQYC

ZmTPPA.3/1-369 GCSSSPSMSASSRRKIEEVLVNG-L-LDAMKSSSPRKKHNL-AFGQDNSPD---EDPAYT

ZmTPPB.1.1/1-367 AVRKKCLQMGAGAGRI------GGW-VESMRASSPTHAKAAAALAAGV------EEERYA

ZmTPPB.1.2/1-384 AVRRKCLQAEVGGGAC--------WGVESMRASSPTHARAAAALAGAGAD----EEEERA

ZmTPPB.1.3/1-370 AVRRKCLQVELGAGAG-LLGGAGAWGVESMRASSPTHARAAAALAAGGGVDVDVDEERAA

ZmTPPB.1.4/1-357 AMSLRYLDLAAAAARS----ASGTW-ADAMRASSPTRSRAA---------------DEFT

ZmTPPB.1.5/1-356 AMSLRYLDLAAAAARS----ASCTW-VEAMRASSPTRSRAAADV------------DELT

ZmTPPB.1.6/1-384 GGEFRSPVAAAANAPPGRTSCTSRV-VEAIRASSPARCPAV---------------DEYD

ZmTPPB.2.1_ramosa3/1-361 DCKKHAAVDLSASGAAVV--GGGPW-FESMKASSPRRAADA----------------EHG

ZmTPPB.2.2/1-327 ------------ARRV----AAGSL-PELVRRHA-----------------------DLD

ZmTPPB.2.3/1-357 GCRLAPAVAAAARQATDDPGAAGSW-PELV---VPRHA-------------------DFD

MOTIF I

ZmTPPA.1/1-388 NWTVNYPSALISFEAISDLAGSKRLALFL**DYDGTL**SPIVDNPENALMSDEMRAAVRHAAS

ZmTPPA.3/1-369 AWLSKCPSALASFKQIVANAQGRRIAVFL**DYDGTL**SPIVDDPDKAFMSPVMRAAVRNVAK

ZmTPPB.1.1/1-367 AWMVKHPSALAMFDQLVAASKGKQIVVFL**DYDGTL**SPIVDDPDAAYMSDTMRRAVRSVAK

ZmTPPB.1.2/1-384 AWMARHPSALGKFERIVAASEGRRIVMFL**DYDGTL**SPIVDDPDAAFMSETMRMAVRSVAK

ZmTPPB.1.3/1-370 SWMARHPSALGRFERIVAAAEGKRIVMFL**DYDGTL**SPIVDDPDAAFMSETMRMAVRSVAK

ZmTPPB.1.4/1-357 AWVRKHPSALGKFEQIASASKGKKVVMFL**DYDGTL**SPIVADPDAAYMSDAMRAAVRDVAK

ZmTPPB.1.5/1-356 AWMRKHPSALGKFEQIASASQGKKVVMFL**DYDGTL**APIVADPDAAYMSDVMRAAVRDVAK

ZmTPPB.1.6/1-384 AWTRKHPSALGSFDQIAAAAKGKRVVMFM**DYDGTL**SPIVADPDMAFMTPEMRAAVRNVAK

ZmTPPB.2.1_ramosa3/1-361 DWMEKHPSALAQFEPLLAAAKGKQIVMFL**DYDGTL**SPIVEDPDRAVMSEEMREAVRRVAE

ZmTPPB.2.2/1-327 DWMEKHPSALAGFESVLAAAEGKQVVMFL**DYDGTL**SPIVKDPDSAVMSEEMRDAVRGVAE

ZmTPPB.2.3/1-357 DWMEKHPSALAAFESVLAAAKGKKIVMFL**DYDGTL**SPIVRDPDSAVMSEEMRDAVRGVAE

MOTIF II

ZmTPPA.1/1-388 LFPTAII**SGR**SRDKVFDFVKLNELYYAGSHGMDIMGP--VRKTTDSNGVECIRSTDVHGK

ZmTPPA.3/1-369 YFPTAIV**SGR**SRKKVFEFVKLTELYYAGSHGMDIVTS-----AAAHATEKC--------K

ZmTPPB.1.1/1-367 HFPTAIV**SGR**CRDKVFEFVKLAELYYAGSHGMDIKGP----AKGSRHT-KA-------KG

ZmTPPB.1.2/1-384 HFPTAIV**SGR**CRDKVFGFVKLAELYYAGSHGMDIKGP--AKASSSRHE-KA-------KA

ZmTPPB.1.3/1-370 HFPTAIV**SGR**CRDKVFEFVKLAELYYAGSHGMDIKGPAAAKASSSSRH-AA-------KA

ZmTPPB.1.4/1-357 HFPTSIV**SGR**CRDKVRNFVALSELYYAGSHGMDIKGP------------SS-------NP

ZmTPPB.1.5/1-356 HFPTAIV**SGR**CRDKVRSFVDLSELYYAGSHGMDIEGP------------SS-------NP

```
ZmTPPB.1.6/1-384          RFPTAIVTGRCIEKVCSFVGLPELYYAGSHGMDIKGP---------NS-KE-------DK
ZmTPPB.2.1_ramosa3/1-361  HFPTAIVSGRCRDKVLNFVKLTELYYAGSHGMDIQGP--AACRQPNHVQQA-------EA
ZmTPPB.2.2/1-327          HFPTAIVSGRCRDKVFNFVKLAELYYAGSHGMDIKGP----TAQSKHT-KA-------KA
ZmTPPB.2.3/1-357          HFPTAIVSGRCRDKVFNFVKLAELYYAGSHGMDIKGP----TAQSKHT-KA-------KA

ZmTPPA.1/1-388            EVNLFQP--ASEFLPMITEVYEKLGESVKDIDGARMEDNKFCVSVHYRNVAEDDYKKVFH
ZmTPPA.3/1-369            EANLFQP--ACEFLPMINEVSKCLVEVTSSIEGARVENNKFCVSVHYRNVAEKDWKVVAG
ZmTPPB.1.1/1-367          GGVLFQP--ASQFLPMIEQVHDSLVEKTKCIPGAKVENNKFCVSVHFRCVDEKSWITLAD
ZmTPPB.1.2/1-384          KGVLFQPATASEFLPMIEAVHERLVETTRSIPGAKVENNRFCVSVHFRCVDEKMWGELWE
ZmTPPB.1.3/1-370          KGVVFQP--ASEFLPMIEEVHERLVQTTRCIPGAKVENNRFCVSVHFRRVDEKMWGELSE
ZmTPPB.1.4/1-357          ESVLCQP--ASEFLPVMDEVYKALVEKTKSTPGAKVEHNKFCLSVHFRCVDEKRWNGLAE
ZmTPPB.1.5/1-356          ESVLCQP--ASEFLPVIDEVYKALVEKTKSTPGAKVENNKFCLSVHFRCVDEKRWNALAE
ZmTPPB.1.6/1-384          TVLLLQP--AREFLPVIDKAYKALVEKTKDTTGARVENNKFCLSVHFRCVDEKSWSSLAE
ZmTPPB.2.1_ramosa3/1-361  AAVHYQA--ASEFLPVIEEVFRTLTAKMESIAGARVEHNKYCLSVHFRCVREEEWNAVNE
ZmTPPB.2.2/1-327          EAVLCQP--ASAFLPVIDEAYRALTARTAPIPGATVENNKFCLSVHFRCVQEEKWRALEE
ZmTPPB.2.3/1-357          GAVLCQP--ARAFLPVIEEVYRALTASTAPIPGATVENNKFCLSVHFRCVQEEKWRALEE

                                                                        MOTIF III
ZmTPPA.1/1-388            RVTAVLEGYPCLRLTHGRKVFEVRPVIDWNKGKAVEFLLESLGL-SESEDVLPIYVGDDR
ZmTPPA.3/1-369            LVKQVLEAFPRLKVTNGRMVLEVRPVIDWDKGKAVEFLLRSLGL-SDSEDVVPIYIGDDR
ZmTPPB.1.1/1-367          MVKSVLKDYPKLKLTQGRMVFEVRPTIKWDKGKALEFLLESLGY-ADCTDVLPVYIGDDR
ZmTPPB.1.2/1-384          SVKGVLREYPRLRLTQGRMVLEVRPTIKWDKGKALEFLLESLGF-AGCTNVLPVYIGDDR
ZmTPPB.1.3/1-370          SVRGVLRGYPKLRLTHGRMVLEVRPSIKWDKGKALEFLLESLGF-ADCSSVLPVYIGDDR
ZmTPPB.1.4/1-357          QVKAVTKDYPKLKLTHGRKVLEIRPSIMWDKGKALEFLLESLGF-ANRSDVLPVYIGDDR
ZmTPPB.1.5/1-356          QVKAVIKDYPKLKLTQGRKVLEIRPSIMWDKGKALEFLLESLGF-ASCSDALPVYIGDDR
ZmTPPB.1.6/1-384          KVKAVLRDFPELELTEGRKVVEVRPSIMWDKGKAVEFLLRSLGFDDDRTNVLPVYIGDDR
ZmTPPB.2.1_ramosa3/1-361  EVRSVLREYPNLKLTHGRKVLEIRPSIKWDKGKALEFLLKSLGY-AGRNDVFPIYIGDDR
ZmTPPB.2.2/1-327          QVRSVLKEYPDLRLTKGRKVLEVRPSIKWDKGNAIQFLLECLGF-ADSNNVFPIYIGDDR
ZmTPPB.2.3/1-357          QVRSVLKEYPDLRLTKGRKVLEIRPSIKWDKGNALQFLLESLGF-AGSNSVFPIYIGDDS

ZmTPPA.1/1-388            TDEDAFKVLKASN--RGFGILVSSIPKESDAFYSLRDPAEVTHSTVATTTNFCFEYFLGQ
ZmTPPA.3/1-369            TDEDAFKVLRERS--CGYGILVSQVPKDTEAFYSLRDPSEVMG----------FLNSLVR
ZmTPPB.1.1/1-367          TDEDAFKVLRKRG--QGVGILVSKHPKDTCASYSLQEPAEVME----------FLLRLVE
ZmTPPB.1.2/1-384          TDEDAFRALRRRGQGQGVGILVSKHPKETSASYSLQEPAEVME----------FLLRLVE
ZmTPPB.1.3/1-370          TDEDAFKVLRRRGQDQGVGILVSKHPKETSASYSLQGPAEVRA-----------------
ZmTPPB.1.4/1-357          TDEDAFKVLRKRG--QGIGILVSKCPKETNASYSLQDPGEVMD----------FLLRLVD
ZmTPPB.1.5/1-356          TDEDAFKVLRKRG--QGVGILVSKCPKETNASYSLQDPGEVMD----------FLLRLVE
ZmTPPB.1.6/1-384          TDEDAFKVLRERG--QGIGILVSKCPKETDATYSLQDPTEVME----------FLVRLGQ
ZmTPPB.2.1_ramosa3/1-361  TDEDAFKVLRNMG--QGIGILVSKLPKETAASYSLSDPAEVKE----------FLRKLAN
ZmTPPB.2.2/1-327          TDEDAFKVLRGMG--QGIGILVSKIPKETSASYSLREPSEVKE----------FLHMLVR
ZmTPPB.2.3/1-357          TDEDAFKVLRNLG--QGIGILVSKIPKETRASYSLREPSEVEE----------FLRKLVS

ZmTPPA.1/1-388            KEN---------------
ZmTPPA.3/1-369            WKKHPL------------
ZmTPPB.1.1/1-367          WERLSKARPKW-------
ZmTPPB.1.2/1-384          WKRLSRLSRTQ-------
```

```
ZmTPPB.1.3/1-370         ------------------
ZmTPPB.1.4/1-357         WKRKSSAAPMVRPRV---
ZmTPPB.1.5/1-356         WKRKSTTTRPPV------
ZmTPPB.1.6/1-384         WNPLRSPSPAARPRGRKQ
ZmTPPB.2.1_ramosa3/1-361 KKGARQP-----------
ZmTPPB.2.2/1-327         SKQR--------------
ZmTPPB.2.3/1-357         WSKESRQRD---------
```

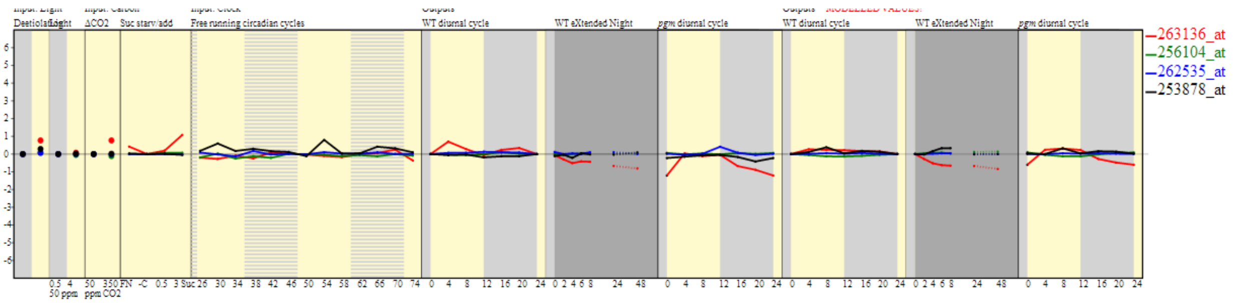

A

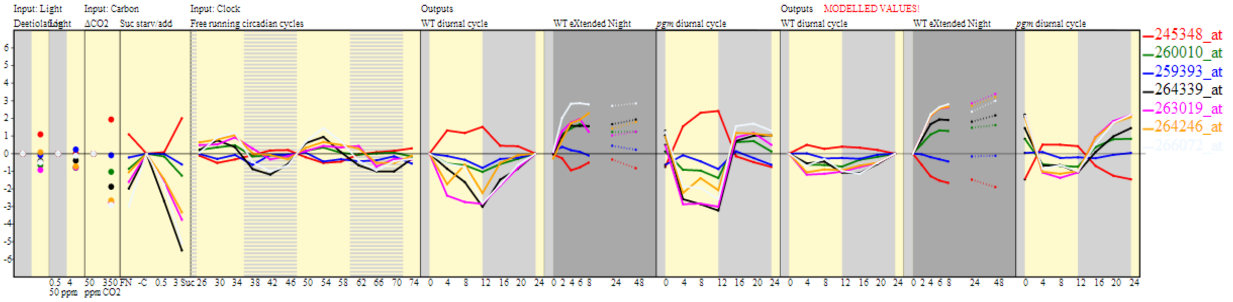

B

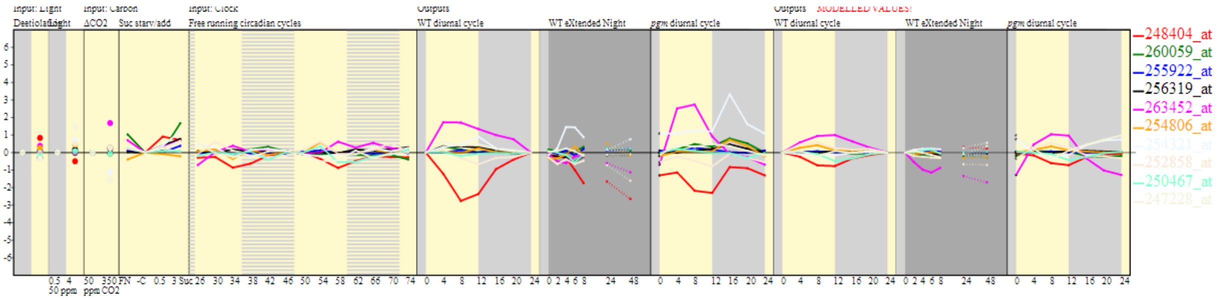

C

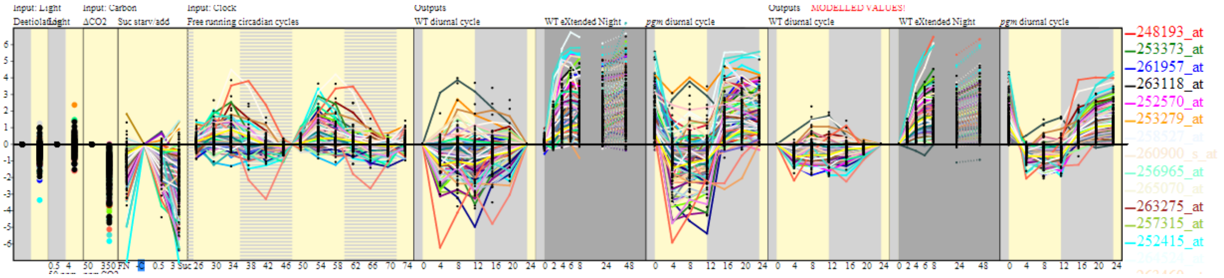

D

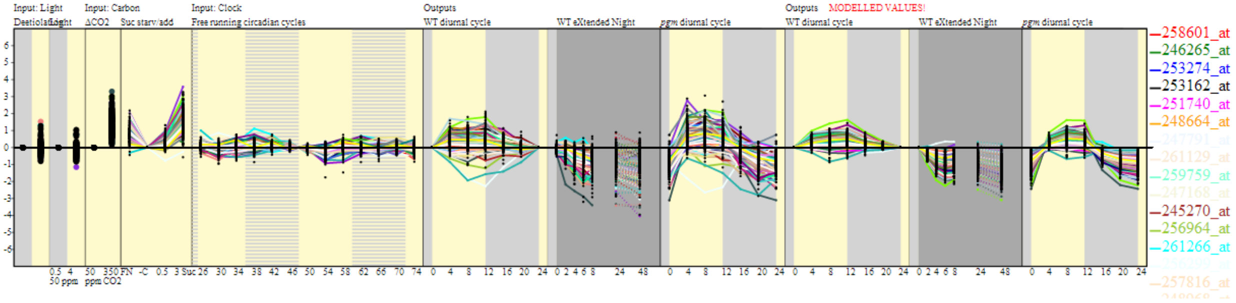

E

Supplemental Figure S3. Expression of Arabidopsis (A)class I TPS genes, (B) class II TPS genes, (C) TPP
SNRK1 (inducible) targets, (E) SnRK1 (repressible) targets in mature leaf tissue throughout the diurna
after 4 h extended night.
